# Statistical approach to analyze wear parameters and worn surface morphology of aluminum – silicon carbide (13%) – graphite composites with and without cryogenic treatment

**DOI:** 10.1038/s41598-026-57887-3

**Published:** 2026-06-17

**Authors:** K. N. Arun Kumar, G. B. Krishnappa, C. S. Thamme Gowda, H. K. Sachidananda

**Affiliations:** 1 Department of Mechanical Engineering, Vidyavardhaka college of Engineering, Mysuru, 570002 Karnataka India; 2 School of Engineering and IT, Manipal Academy of Higher Education, Dubai, 345050 UAE

**Keywords:** Aluminium matrix composites, AL-SiC-Graphite, Hybrid MMC, Cryogenic treatment, Sliding wear, Taguchi approach, ANOVA, Engineering, Materials science

## Abstract

Recent technology requires materials with unusual combinations of properties to meet specific needs. Composite materials are gaining more importance due to high toughness, stiffness, specific strength, strength to weight ratio etc. Hybrid composite materials are in more demand with varying reinforcement which enhances mechanical properties. The present work is aimed at reinforcing AL6061 alloy with 13% Silicon carbide and varying % of graphite (1%, 2% and 3%). The hybrid composites are fabricated using bottom pouring stir casting machine. The fabrication of specimens is done as per ASTM standards. The wear characteristics of Al–SiC (13%)-Graphite composites, with and without cryogenic treatment, were investigated using a statistical optimization approach based on the Taguchi method. The multiple regression equation obtained using ANOVA correlates the evaluation of the wear of the HYBRID combination of both untreated and cryogenically treated (CT) specimens with a reasonable degree of approximation. Cryogenic treatment resulted in a reduction in wear scar depth and coefficient of friction under the tested conditions. The Al 6061/13%SiC/2%Gr is best among all the combinations studied.

## Introduction

The physical and mechanical behaviour of some metals will enhance when it is subjected to thermal treatments. Thermal treatments include heat treatment and cold treatment. Cold treatment is also called sub-zero treatment. In recent years, considerable attention has been directed toward cold treatment for enhancing the properties of metals and this process is typically categorized as cold treatment, carried out at temperatures as low as that of dry ice (−78.5^0^ or 194.7 K). Important cold treatment is the cryogenic treatment. Cryogenic treatment involves cooling specimens to cryogenic temperatures, maintaining them at that level for a specified duration, and subsequently returning them to room temperature at a controlled rate. This treatment has been shown to enhance the properties of monolithic metals, alloys, steels, and metal matrix composites (MMCs). The improvements are attributed to microstructural modifications, including grain refinement, transformation in crystal structure, and stabilization of the material’s chemical composition under deep cryogenic conditions. Post-treatment analysis reveals visible changes in the material, with metallurgical examinations confirming a more uniform, refined, and densely packed microstructure. Hence it is imperative that study on wear properties of cryogenically treated aluminium metal matrix composites would make a good contribution to the research society.

 Lulay et al.^1^ have studied the effect of cryogenic treatment on aluminium 7075 alloy. It was observed that there was no statistically significant effect on basic properties after 2 h of cryogenic treatment but a very minor improvement in the hardness value after 48 h. Kim et al.^2^ have conducted an experiment to see how cryogenic therapy affected nickel-titanium endodontic equipment. Increased microhardness was reported because of the cryogenic treatment; however, this increase was not identified clinically. There was no discernible change in the composition of the elemental or crystalline phases.

 Farhani et al.^3^ have reviewed and explained various mechanisms which involve in the process of cryogenically treated and showed that the cryogenically treated metals increase the wear resistance. They concluded that better results are due to effective cryogenically treated that involves correct soaking time and soaking temperature. It is also important to have a controlled programmable device. Joel Hemanth et al.^4^ examined the fracture characteristics of aluminum alloy composites reinforced with nano-ZrO₂, which were solidified using cryogenic techniques. The nano ZrO_2_ concentration ranges from 3 to 15 wt% in 3 wt% increments. It was discovered that a chill thickness of 25 mm and 12% ZrO_2_ improves strength, hardness, and fracture toughness while reducing ductility slightly. Ajit et al.^5^ have examined the cryogenic treatment of steels in a deep-freezing environment (−196 °C) for 24 h and then gradually warmed to ambient temperature. It was observed, that cryogenically treated changes microstructure due to precipitation and results in retained austenite turned to martensite. It was also observed that the carbide structures were refined, and stress was relieved.

 Panchakshari et al.^6^ have experimented with the cryogenic treatment of Al6061-Al_2_O_3_ matrix composites to see how it affects microstructure and microhardness. Nonferrous metals that have been cryogenically treated have a longer wear life and are more durable. XRD analysis reveals that cryogenic treatment affects the intensity of diffraction peaks corresponding to various crystal planes in metal matrix composites (MMCs), confirming enhanced hardness in Al-Al₂O₃ composites. Candane et al.^7^ examined the grain structure, and wear of AISI M35 HSS under cryogenic and conventional heat treatments. Their findings revealed that specimens subjected to 24 h of cryogenic treatment exhibited a very minor increase in wear and hardness value. Cryogenic cold treated samples revealed fine carbide precipitates with sizes ranging from 0.3 to 0.5 microns. No improvement was observed in toughness after cryogenic treatment.

 Nageswara Rao et al.^8^ have investigated hardness value and tensile properties of Al6061 alloy after cryo-treatment in liquid nitrogen. Both properties were enhanced for the forged alloy which can be attributed to increase in dislocation density. Wen-da ZHANG et al.^9^ have studied the tensile properties of 3104 aluminum alloy. This Al alloy was processed by homogenization treatment (580° C) for 10 h and deep cryogenic treatment (−196° C) for 24 h. Tensile properties were studied for four different conditions namely homogenization treatment (HT), deep cryogenic treatment (DCT), HT-DCT, and DCT-HT. Specimens exposed to deep cryogenic treatment combined with subsequent heat treatment (DCT–HT) exhibited significant improvements in tensile strength, yield strength, and ductility. G Elango et al.^10^ have investigated both LM25 alloys and LM25 reinforced with SiC upon cryogenic treatment. The microstructure and hardness are studied for different cryogenic treatment duration (1, 5, 10, 20, 30, 40, and 50 h). Cryogenically treated MMC specimens produced superior outcomes than untreated MMC specimens. Doo-Hwan Park et al.^11^ have conducted an experiment to study the mechanical properties for AA6061, AA6082, AA5052, and AA5086 on cryo-treatment. It was observed that AA6061 exhibits higher tensile strength, and yield strength after cryogenic treatment. The specimen’s fracture surface changed to a cup-and-cone fracture for AA5052 and AA6082).

 Volker Franco Steier et al.^12^ have studied the wear and microstructure of an Al6061 alloy on deep cryo-treatment. DCT enhances the wear and other properties of Al alloy. DCT shows a marginal improvement in grain structure. Al-Alkawi Hussain Jasim Mohammed et al.^13^ have conducted an experiment on Al7075/Al_2_O_3_ particles to examine the mechanical properties on cryogenic treatments. The composite Al7075 with varying wt% (0.2, 0.4, 0.6, 0.8, and 1), of Al_2_O_3_ are fabricated using stir casting method. The cryogenic treatment was done for 24 h. Deep cryogenic treatment was found to enhance the properties of the composites for Al alloy reinforced with 0.2 wt% Al₂O₃.

 G Ramanjaneya Yadav et al.^14^ have conducted an experiment to know the compressive strength of Al-Ci alloys on effect of cold treatment. The specimens were fabricated by varying the copper percentage (1.5–3.0, and 4.5 wt%) in the Aluminium-Copper Alloy by the metal casting process. The produced alloy specimens were cold treated with liquid nitrogen as cold chamber which will be soaked for about 6, 8, and 10 h, respectively. The duration of cryogenic treatment was found to boost the Al-Cu alloy’s compression strength. B. V. Padmini et al.^15^ explored the wear characteristics of aluminum alloys subjected to cryogenic conditions, specifically assessing the impact of a 64-hour deep cryogenic treatment (DCT) on Al 2024, Al 6082, and Al 7075 alloys. Their study reported that DCT specimens exhibited superior wear resistance compared to untreated counterparts.

 Jianzhong Zhou et al.^16^ investigated the impact of a 24-hour cryogenic treatment on 2024-T351 aluminum alloy, finding that the process enhanced the refinement and uniformity of secondary phase distribution. Since grain boundaries are strongly associated with these phases, the treatment also promoted the formation of more uniform grains. Compared to the untreated specimens, cryogenically treated specimens showed improvement in tensile properties, and fatigue life. A. Jeyachandran et al.^17^ studied the impact of cryogenic treatment on the microstructural properties and wear behaviour of Al6061 hybrid metal matrix composites containing 2.5 wt% SiC and 2.5 wt% TiO₂. Their research indicated that deep cryogenic treatment substantially enhanced the hardness and wear resistance of the Al6061–SiC–TiO₂ composites.

 Prasanna Venkatesh et al.^18^ examined the wear characteristics of Al/fly ash composites subjected to cryogenic treatment. Results indicated that an extended cryogenic soaking period led to a reduction in wear rate and Pavithra A et al.^19^ have examined the cryogenically treated specimens on AL alloy 356 by stir-casting method. The tensile, hardness, and corrosion test was conducted on Aluminum alloy 356 reinforced with SiC (4, 6, 8, and 10 wt%). The cryogenic soaking takes place for around 72 h. The 6 wt% of SiC reinforcement shows an improved microstructure with grain refinement and grain modification. Heat treated specimens show improve mechanical properties compared to cryogenically treated specimens.

 Pankaj Sonia et al.^20^ investigated the effect of cryogenically treated on the mechanical properties and microstructure of Al 6082 alloy by varying the soaking duration which varies from 10 min to 24 h. Their results showed a reduction in grain size with increasing soaking time, along with improvements in tensile strength and hardness, the latter attributed to strain hardening. Similarly, M. K. Chaanthini et al.^21^ examined Al 2014 alloy under shallow cryogenic treatment (SCT) for soaking intervals of 3–30 h. They reported that ultimate tensile strength, elongation, fracture toughness, and hardness reached peak values at 18 h of SCT, fluctuated between 18 and 24 h, and again attained maxima at 30 h.

 J.D. Darwin et al.^22^ have analysed the Taguchi method for improving wear resistance by Cryotreatment of 18% Cr martensitic structured steel. The four factors were considered in the study cooling rate (0.5, 1, and 1.5 ^o^ centigrade/min), soaking temperature (−120, −150, and − 184° centigrade), soaking period (12, 24, and 36 h), and tempering temperature (200, 250 and 300 °C). It was observed that soaking temperature (−184^o^ centigrade) was the most significant factor, followed by a soaking period (36 h), cooling rate (1^o^ centigrade/min), and tempering temperature (250^o^ centigrade). Chennakesava Reddy et al.^23^ used the statistical approach to optimise the parameters of Aluminum reinforced with Silicon Carbide. The study considered different aluminum alloys (Al 6061, Al 6063, and Al 7072), reinforcement fractions (12%, 16%, and 20%), and SiC particle sizes (10, 15, and 20 μm). Results indicated that Al 6061 exhibited superior ultimate tensile and bending strength compared to Al 6063 and Al 7072. Similarly, Mohamed Zakaulla et al.^24^ applied the Taguchi DOE approach to optimize wear parameters of Aluminum 6061 alloy reinforced with SiC and Gr which were coated by copper. Employing an L27 orthogonal array to examine the effects of load (10–40 N), sliding speed (0.42–1.68 m/s), and sliding distance (750–3000 m), the study identified load as the primary factor influencing specific wear rate, followed by sliding speed and then sliding distance.

 Divya Sadhana et al.^25^ employed the Taguchi DOE approach to optimize the wear parameters of LM25–fly ash composites, with LM25 aluminum reinforced with fly ash. The experiments were designed using a 27 array, considering four factors: load (15, 30, and 45 N), (1, 1.5, and 2 m/s) sliding speed, (500, 1000, and 1500 m) sliding distance, and reinforcement content (3, 6, and 9 wt%). The analysis revealed that load had the strongest statistical impact, with sliding speed, reinforcement, and sliding distance contributing progressively lesser effects. Sathish et al.^26^ have experimented with optimizing the wear parameters of Al7050-SiC composites using the Taguchi method. It was observed that the sliding distance was the most influenced parameter on the wear. Microstructure images showed uniform distribution of SiC reinforcement on base alloy, and it provides resistance to wear.

 S. Arunkumar et al.^27^ have analysed the Taguchi method to optimize the wear parameters of Al7075/TiC/Gr-/Al_2_O_3_ hybrid composites. The four factors with levels TiC, Gr (6 and 9 wt%), Al_2_O_3_, and load were considered. It was observed that TiC (6%) yields the better results, followed by Al_2_O_3_ (7%), load (20 N), and Gr (9%). Nenad Miloradovic et al.^28^ have experimented with optimizing wear parameters and Gr (3 and 5 wt%), load, and sliding speed were considered. The findings indicated that load was the dominant factor affecting specific wear rate, with sliding speed and graphite content having lesser impacts. Of the materials tested, Zn-Al-10% SiC-5% Gr hybrid composites demonstrated superior wear resistance relative to Zn-Al-10% SiC-3% Gr. Microstructural analysis verified a consistent and uniform distribution of SiC and graphite reinforcements within the base alloy. Zeng et al.^29^ has studies gear wear research by proposing a dynamic wear evolution incorporating real-time variations in contact within modified gear rack systems. They stressed on conventional wear models which assume constant contact condition fail to capture the progressive alteration of meshing interfaces under operational conditions. So, they are integrating time-varying contact stress, sliding velocity and evolving surface geometry and demonstrated how wear modifies local contact conditions in a feedback-driven manner. They concluded that localized stress redistribution accelerates wear in critical regions thereby influencing transmission performance. Zheng et al.^30^ studied a hybrid intelligent modelling framework to predict friction and wear behaviour in a copper-free resin brake material. He integrated particle swarm optimization (PSO) and flower pollination algorithm (FPA) with a backpropagation neural network (BP) to enhance prediction accuracy and convergence stability. They captured the nonlinear relationships between operating parameters and tribological responses to predict friction coefficient and wear rate under varying conditions. They concluded that hybrid optimization approach reduces the risk of local minima typically associated with conventional BP models, thereby improving robustness.

Recent advances in tribology have increasingly shifted from conventional static wear characterization toward dynamic and predictive modelling frameworks that capture the evolution of contact conditions during sliding interactions. For instance, studies on dynamic wear evolution have demonstrated that wear processes are governed by time-dependent variations in contact stress, sliding velocity, and surface morphology, which are not adequately represented in traditional steady-state models^29^. Similarly, hybrid intelligent modelling approaches integrating optimization algorithms with machine learning techniques have been employed to predict friction and wear behaviour under varying operational conditions, revealing complex nonlinear relationships between process parameters and tribological responses^30^. Despite these advances, most existing studies on aluminum-based metal matrix composites, particularly Al6061 reinforced with SiC and graphite, remain limited to experimental and statistical optimization approaches without integrating evolving wear mechanisms or predictive modelling perspectives. Furthermore, although cryogenic treatment has been widely reported to enhance wear resistance through microstructural refinement and improved phase stability, its interaction with hybrid reinforcement systems and its influence under varying tribological conditions remain insufficiently understood. Prior studies have predominantly focused on single-reinforcement systems or isolated property evaluation, with limited attention to multi-reinforcement hybrid composites and their statistically optimized wear behaviour.

Therefore, a clear research gap exists in systematically analysing the wear behaviour of Al6061–SiC–Gr hybrid composites under cryogenic treatment by integrating statistically robust experimental design with insights from emerging wear modelling paradigms. The present study addresses this gap by employing a Taguchi-based design of experiments combined with ANOVA and regression modelling to evaluate the influence of process parameters on wear scar depth and coefficient of friction, while also interpreting the results in the context of evolving wear mechanisms and surface morphology. This approach provides both experimental optimization and a pathway toward bridging conventional statistical analysis with emerging predictive frameworks.

Previous studies have demonstrated that ceramic and solid-lubricant reinforcements can effectively tailor the mechanical and tribological properties of aluminium matrix composites. The type and composition of reinforcements play a very important role in governing the mechanical performance of aluminium-based composites. Microstructural analyses further reveal that stir casting generally ensures a proper mixing of reinforcement without much porosity in the final specimens. Hybrid reinforcement systems generally exhibit superior performance compared with single-reinforcement composites. Also, the cryogenic treatment enhances the mechanical and thermal characteristics of Aluminium alloys although it may reduce impact strength. While more research work has been done on Al alloys reinforced with either SiC or Gr individually, limited work is available on Al6061-SiC-Gr hybrid composites. Moreover, the reinforcement weight fractions have not been fully optimized, and the effect of cryogenic treatment on Al6061-SiC-Gr composites remain largely unexplored. This highlights a clear scope for further investigation in this area.

Recent advances in materials engineering have increasingly incorporated data-driven and machine learning-based approaches for predicting material performance and optimizing process parameters. For instance, machine learning models such as random forest regression have been successfully employed to capture complex nonlinear relationships between composition, processing conditions, and performance characteristics, thereby improving predictive accuracy in advanced material systems^31^. However, such approaches generally require large datasets and extensive computational resources, which may not be feasible in experimental studies involving hybrid metal matrix composites with limited observations. In this context, statistical design of experiments methods, such as the Taguchi approach combined with ANOVA and regression modelling, remain highly effective for identifying significant parameters and optimizing performance with a reduced number of experimental trials. Therefore, the present study adopts a Taguchi-based framework to systematically analyse wear behaviour while providing a reliable and resource-efficient alternative to data-intensive predictive models.

Although significant research has been conducted on aluminium-based composites reinforced with SiC and graphite, most existing studies have primarily focused on either single-reinforcement systems or conventional experimental investigations without integrating advanced statistical optimization techniques. In addition, while cryogenic treatment has been reported to improve mechanical and tribological properties of aluminium alloys, its combined effect with hybrid reinforcement systems and optimized process parameters remains insufficiently explored. Furthermore, recent developments in tribological research emphasize the importance of linking process parameters, microstructural evolution, and performance characteristics; however, such integrated analyses are limited for Al6061-SiC-Gr hybrid composites. Therefore, the novelty of the present study lies in the combined application of Taguchi-based statistical optimization and cryogenic treatment to systematically evaluate the wear behaviour of Al6061-SiC-Gr hybrid composites. The study further establishes a comprehensive relationship between process parameters, surface morphology, and tribological performance, thereby addressing an important gap in the current literature.

## Materials and Methodology

### Materials

**Fig. 1:**
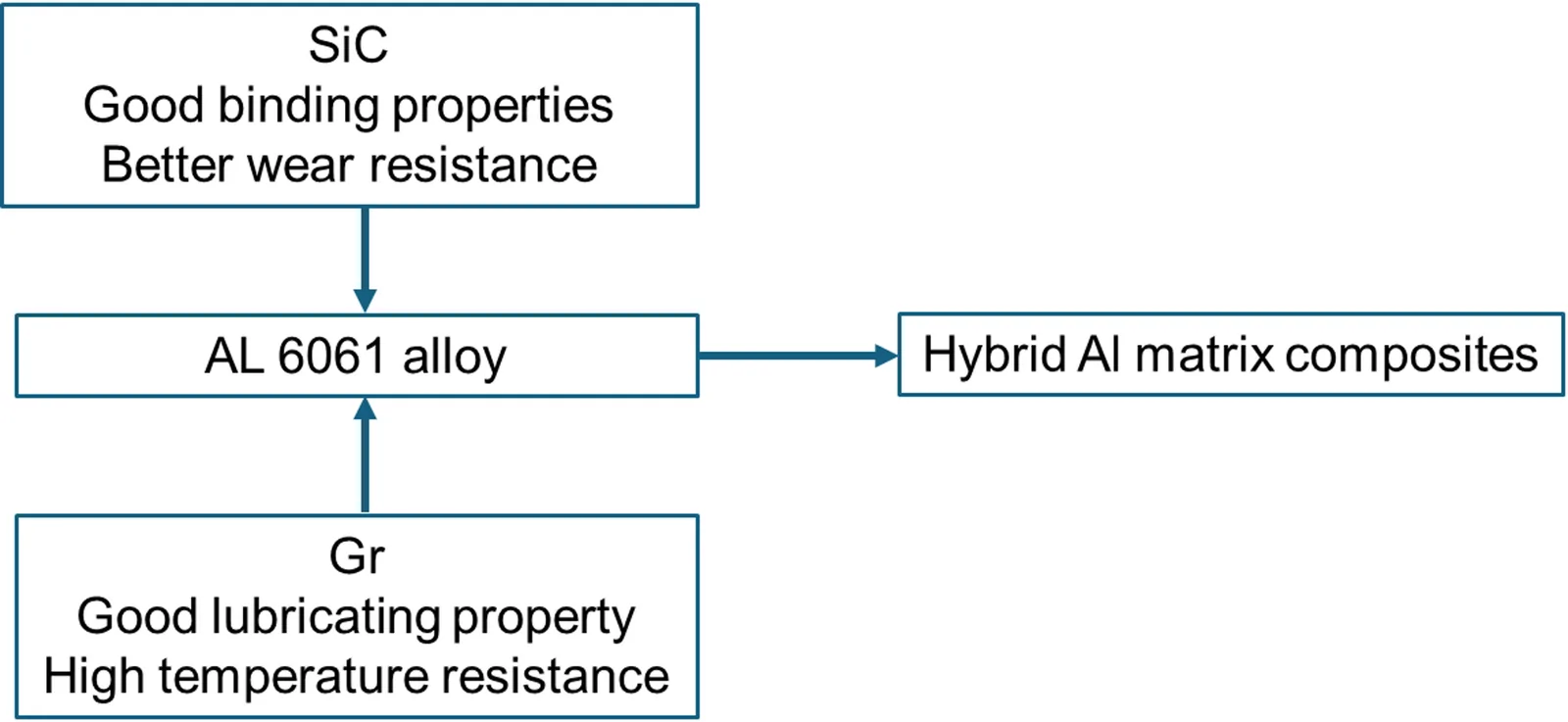
Schematic diagram illustrating the fabrication process of Al6061–SiC–Gr hybrid metal matrix composites using stir casting.

**Fig. 2:**
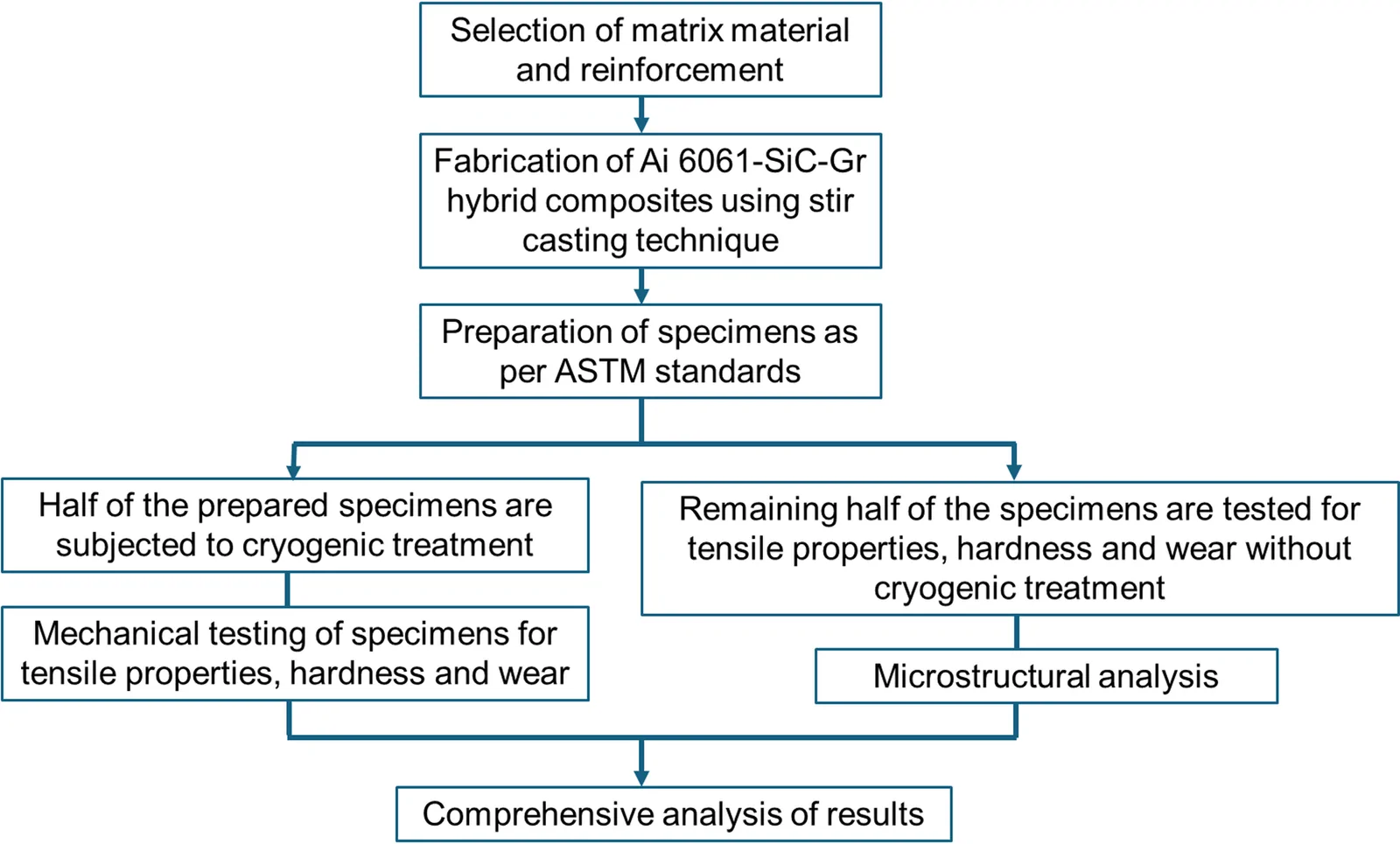
Methodology flowchart illustrating experimental design, fabrication of Al6061–SiC–Gr composites, cryogenic treatment, wear testing using pin-on-disc, and subsequent analysis using Taguchi method, ANOVA, and SEM.

The raw material used is Aluminium alloy - Al 6061 alloy. The Al-6061 alloys have been generally utilized due to their dominating mechanical properties and better formability. The reinforcement material is Silicon carbide (micro powder) and Graphite (micro powder). To improve the characteristics of composites, silicon carbide (SiC) and Graphite (Gr) can be used as reinforcement in the form of particles, whiskers, or Fibers. SiC improves the composites overall strength, as well as corrosion and wear resistance. Tables 1 and 2, and 3 gives the properties of Al6061, SiC and Gr. Figures 1 and 2 shows the material combination and methodology flow chart used for the present study. Important cryogenic fluids and their boiling points are listed in Table 4.

**Table 1:** Physical, mechanical, and thermal properties of Al6061 alloy.

Property	Value	Unit
Density (ρ)	2.70	g/cm^3^
Melting point	582–652	°C
Young’s modulus (E)	68	GPa
Tensile strength (σ_t_)	124–290	MPa
Elongation (ε) at break	12–25	%
Poisson’s ratio (ν)	0.33	
Brinell hardness	30–95	HB
Thermal conductivity	170	W/m·K

**Table 2:** Physical and mechanical properties of silicon carbide (SiC).

Property	Value	Units
Average particle size	40–50	µm
Molecular formula	SiC	
Purity	99	%
Molecular weight	40.10	g/mol
Colour	Grey	
Density	3.2	g/cm^3^
Melting point	2730	^o^ C
Poisson’s ratio	0.34	
Elastic modulus	410	GPa
Thermal conductivity	490	W/(m·K)

**Table 3:** Physical, mechanical, and thermal properties of graphite.

Average Particle size	40–50 μm
Molecular formula	C
Purity	99%
Molecular weight	12.01 g/mol
Colour	Black/Dark grey
Density	1.8 g/cm^3^
Solubility	Insoluble in water
Melting point	3652^o^ C
Poisson’s ratio	0.14
Elastic modulus	21 GPa
Thermal conductivity	6 W/m-K

**Table 4:** Important cryogenic fluids and their boiling points.

Fluid	Boiling point ^o^ C	Type of gas
Argon	−186 (87 K)	Inert
Helium	−269 (4 K)	Inert
Hydrogen	−253 (20 K)	Flammable
Methane	−161 (112 K)	Flammable
Nitrogen	−196 (77 K)	Inert
Oxygen	−183 (90 K)	Oxidizer

### Fabrication

The stir casting technique was employed for the fabrication of Al6061-SiC-Gr hybrid metal matrix composites due to its suitability for particulate reinforcement and cost-effectiveness. The Al6061 alloy was melted in a resistance furnace at a temperature of approximately 750–780 °C and held for 20–30 min to ensure thermal homogeneity and adequate fluidity. Prior to incorporation, SiC and graphite reinforcements were preheated to approximately 400–500 °C to remove surface moisture, improve wettability, and minimize thermal mismatch. The preheated reinforcements were gradually introduced into the molten alloy under continuous stirring. Mechanical stirring was carried out using a graphite-coated steel impeller at a speed of approximately 400–600 rpm for 8–10 min to ensure uniform dispersion of reinforcement particles. The impeller, with a four-blade configuration, was positioned at approximately two-thirds depth of the molten metal to generate a stable vortex and enhance particle incorporation.

To reduce gas porosity and improve casting quality, degassing was performed using hexachloroethane tablets prior to pouring. The molten composite slurry was then poured through a bottom-pouring stir casting setup into a preheated metallic mould. The mould temperature was maintained at approximately 200–300 °C to prevent premature solidification and ensure defect-free casting. The casting was carried out using a metallic mould with five cavities, each having a diameter of 20 mm and a height of 300 mm, to ensure consistent specimen geometry.

**Fig. 3:**
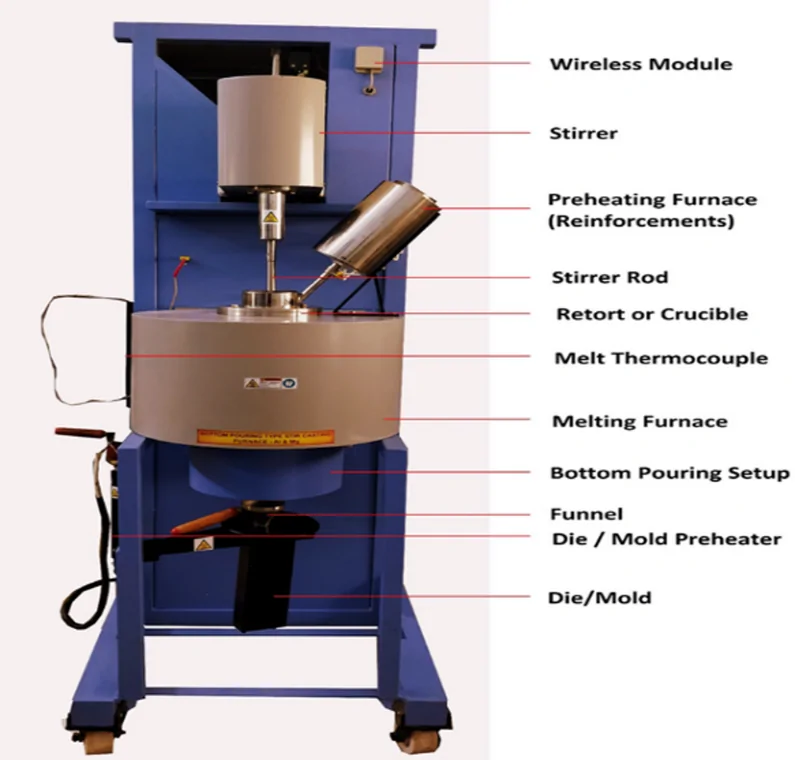
Bottom-pouring stir casting apparatus used for fabrication of Al6061-SiC-Gr hybrid composites.

**Fig. 4:**
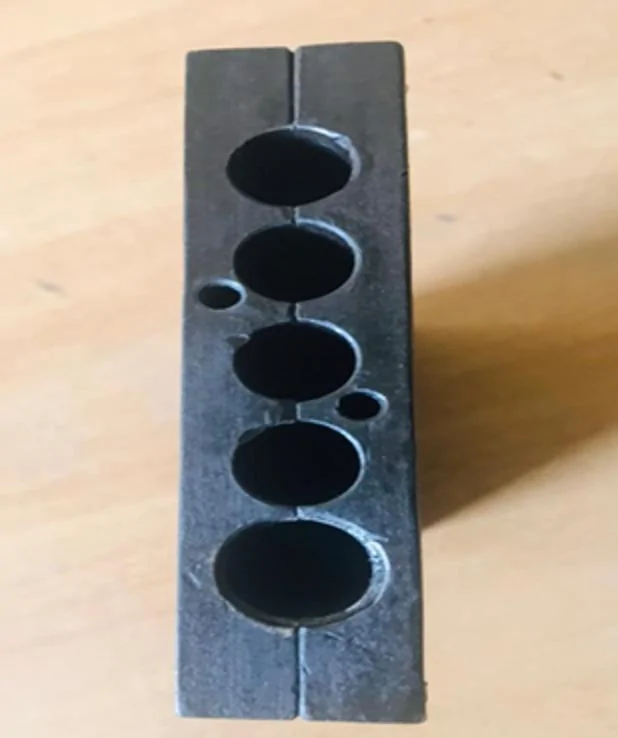
Casting mould box with five cavities of 20 mm diameter and 300 mm height used for specimen preparation.

**Fig. 5:**
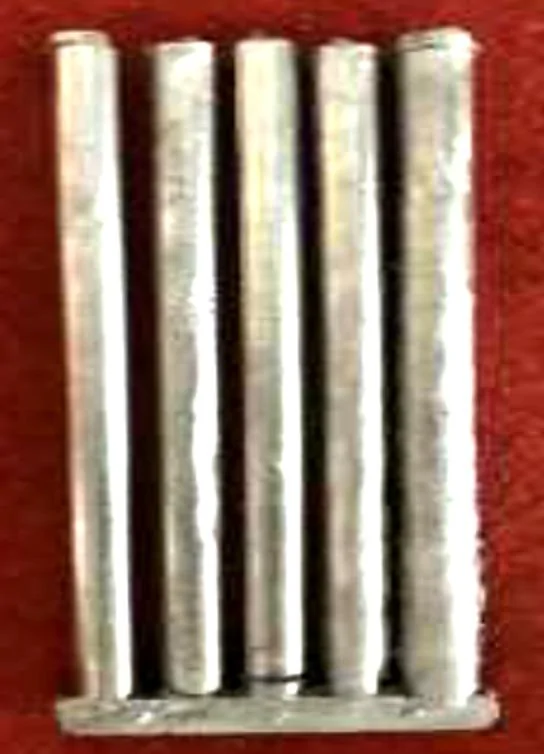
As-cast Al6061-SiC-Gr hybrid composite specimens obtained after stir casting.

The selected reinforcement fraction was maintained below 20 wt% to facilitate effective mixing and minimize particle agglomeration. The cast specimens were allowed to cool under ambient conditions and subsequently machined as per ASTM standards. Figures 3, 4 and 5 illustrate the stir casting setup, mould configuration, and as-cast composite specimens. These controlled processing parameters, including melting temperature, stirring speed, reinforcement preheating, and degassing, play a critical role in achieving uniform particle distribution, minimizing porosity, and enhancing interfacial bonding, which directly influence the tribological performance of hybrid metal matrix composites^32^.

### Mechanical testing

**Fig. 6:**
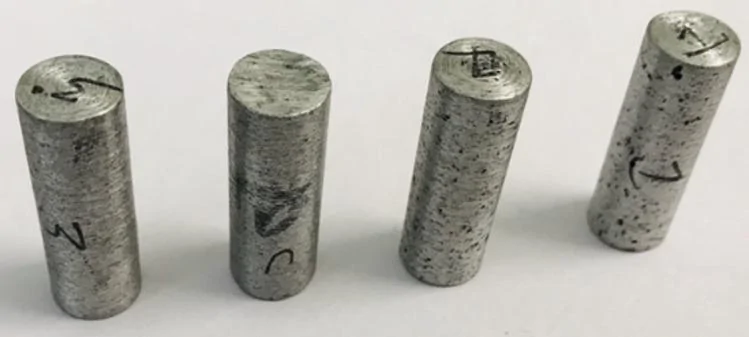
Wear test specimens prepared in accordance with ASTM G99.

All specimens were sectioned using a power hacksaw machine in accordance with ASTM standards. Wear specimens were tested with respect to ASTM G99 standards. Testing of specimens for wear properties such as weight loss, wear in microns, and coefficient of friction is done using wear testing machine which is pin on disc type. A stationary spherical pin will be in touch with the revolving disc for a certain load in a pin on disc wear testing system. The spherical pin (10 mm in diameter) is loaded against a rotating disc with a track diameter of 140 mm using a deadweight loading system. The as-cast HAMC are machined as per ASTM G99 standard. The machined HAMC specimens have been finished using 200, 600, 800, and 1000 grid emery sheets. The HAMC specimens are cleaned using acetone before the test. The wear test is conducted for HAMC specimens to determine wear properties. Figure 6 shows the wear specimens machined as per ASTM G99 standards.

### Cryogenic treatment of prepared specimens

Cryogenic treatment is a controlled thermal process involving cooling to cryogenic temperature, soaking for a specified duration, and gradual return to ambient conditions. In the present study, cryogenic treatment was carried out using liquid nitrogen at approximately − 196 °C following a controlled thermal cycle. The specimens were gradually cooled from room temperature to − 196 °C at an estimated rate of 1–2 °C/min to minimize thermal shock and prevent microcracking. The cooling phase required approximately 3–4 h to reach the target temperature. Once stabilized, the specimens were soaked at − 196 °C for 24 h to ensure uniform temperature distribution and to facilitate microstructural stabilization.

**Fig. 7:**
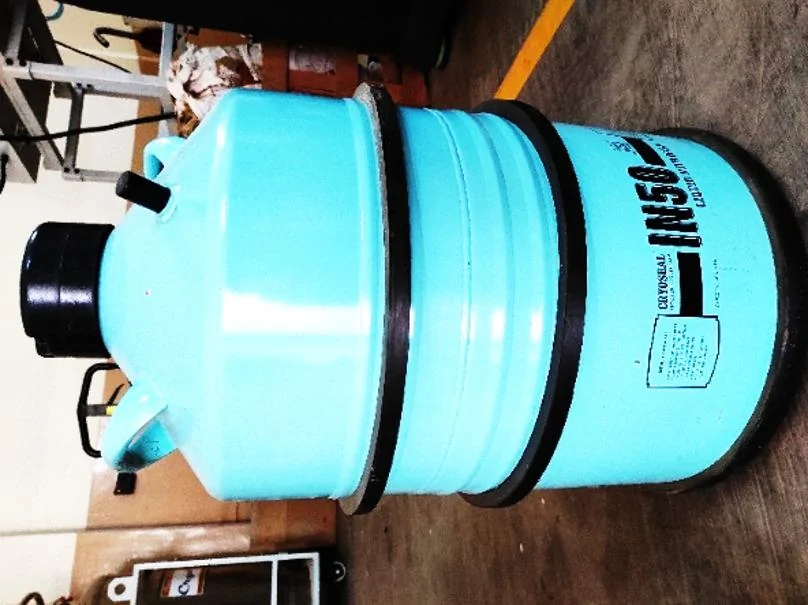
Liquid nitrogen storage container used for cryogenic treatment of specimens.

**Fig. 8:**
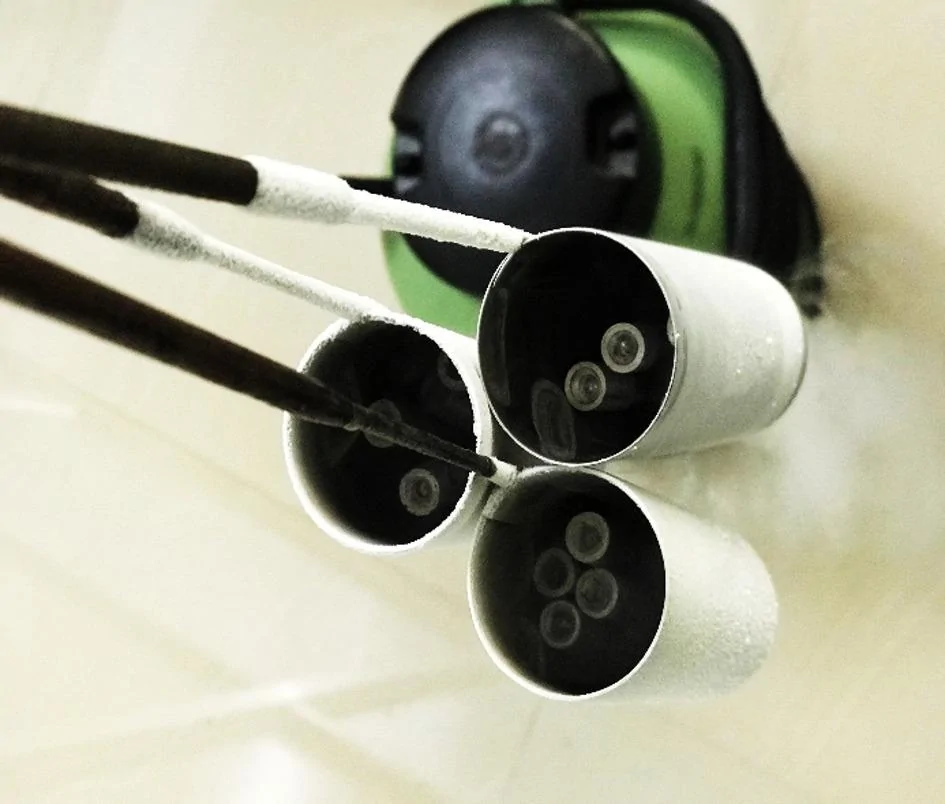
Specimens placed in the canister after removal from the liquid nitrogen container following cryogenic treatment.

**Fig. 9:**
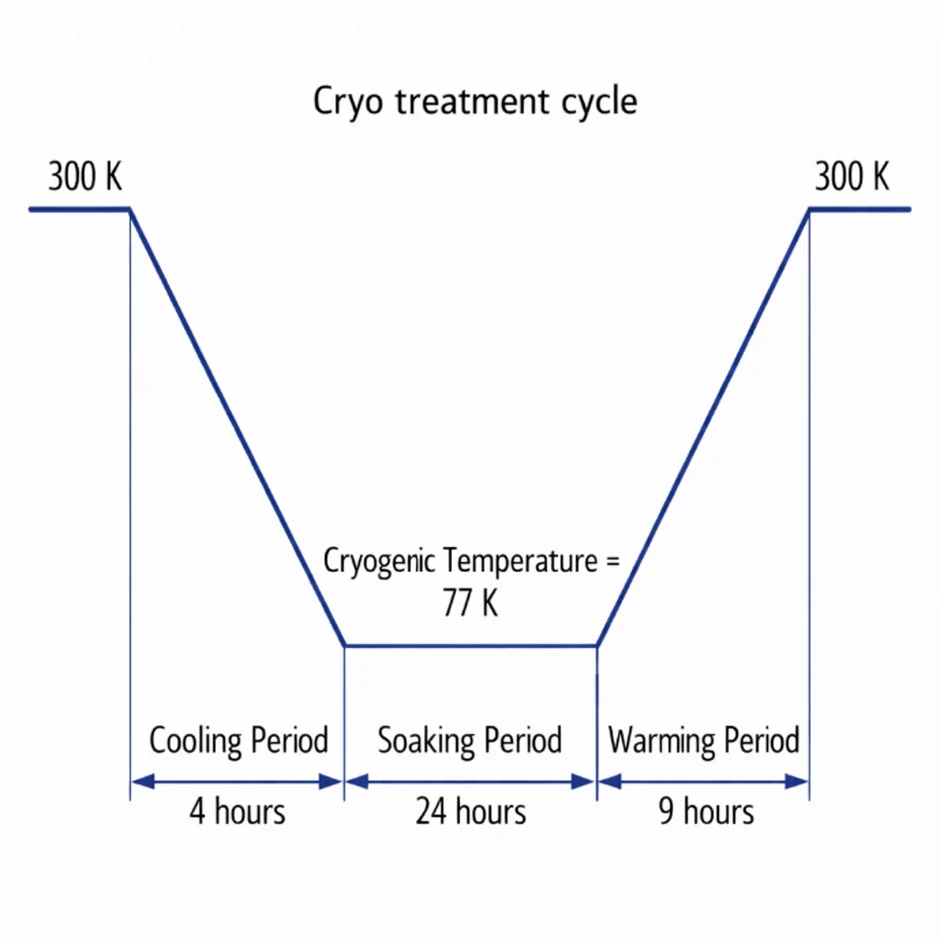
Temperature-time profile of the cryogenic treatment cycle showing cooling, soaking, and heating stages.

Following soaking, the specimens were returned to room temperature through controlled heating at a rate of approximately 0.5–1 °C/min, requiring about 8–10 h. This gradual warming minimizes residual thermal stresses and prevents distortion or microstructural damage. From a metallurgical perspective, cryogenic treatment induces microstructural modifications that contribute to improved tribological performance. These include refinement and redistribution of secondary precipitates, increased dislocation density, and reduction of residual stresses within the matrix. The interaction between refined precipitates and dislocations enhances resistance to plastic deformation, while reduced residual stresses improve dimensional stability and wear resistance. In hybrid metal matrix composites, such microstructural changes also enhance interfacial bonding and load transfer efficiency between the matrix and reinforcement phases, thereby contributing to improved wear behaviour. These mechanisms are consistent with recent findings reported for cryogenically treated aluminium alloys^33^. Figures 7, 8 and 9 illustrate the cryogenic setup, specimen placement, and the temperature–time profile of the treatment cycle. Such improvements in interfacial stability and load transfer are also supported by recent studies on advanced ceramic-reinforced systems, where microstructural refinement plays a critical role in enhancing tribological performance^34^.

## Results and discussion

While recent studies have explored machine learning-based predictive models for wear and material performance, the present study employs a Taguchi-based statistical approach due to its effectiveness in handling multi-parameter optimization with limited experimental data. The Taguchi method is a well-established statistical optimization technique that integrates experimental design, analysis, and parameter optimization to identify optimal process conditions. Its primary objective is to minimize output variability in the presence of noise factors, thereby ensuring robust performance while reducing experimental cost and effort.

In the present study, the Taguchi method was applied to design experiments for abrasive wear analysis, enabling the identification of optimal parameter combinations with a minimal number of trials. Taguchi’s design approach utilizes orthogonal arrays to systematically arrange process parameters and their levels, allowing efficient exploration of factor effects without requiring all possible experimental combinations. The Taguchi L16 orthogonal array was employed to reduce the number of experimental runs while maintaining statistical efficiency.

Due to the adopted design of experiments approach, each experimental condition was conducted once and replication was not performed. While this approach is consistent with Taguchi methodology and enables efficient parameter optimization, it limits the estimation of experimental variability, pure experimental error, and confidence intervals associated with the measured responses. Therefore, the statistical findings should be interpreted within the context of this limitation.

Analysis of variance (ANOVA) was conducted at a 90% confidence level (*p* ≤ 0.1). This threshold was adopted due to the limited number of experimental runs in the Taguchi L16 design, where the use of a stricter confidence level (e.g., 95%) may reduce sensitivity in detecting significant factors. Although a 90% confidence level is commonly employed in engineering optimization studies involving constrained experimental datasets, it represents a less stringent criterion than the conventional 95% confidence level and therefore constitutes an additional limitation of the present statistical analysis.

### Plan of experiments

**Fig. 10:**
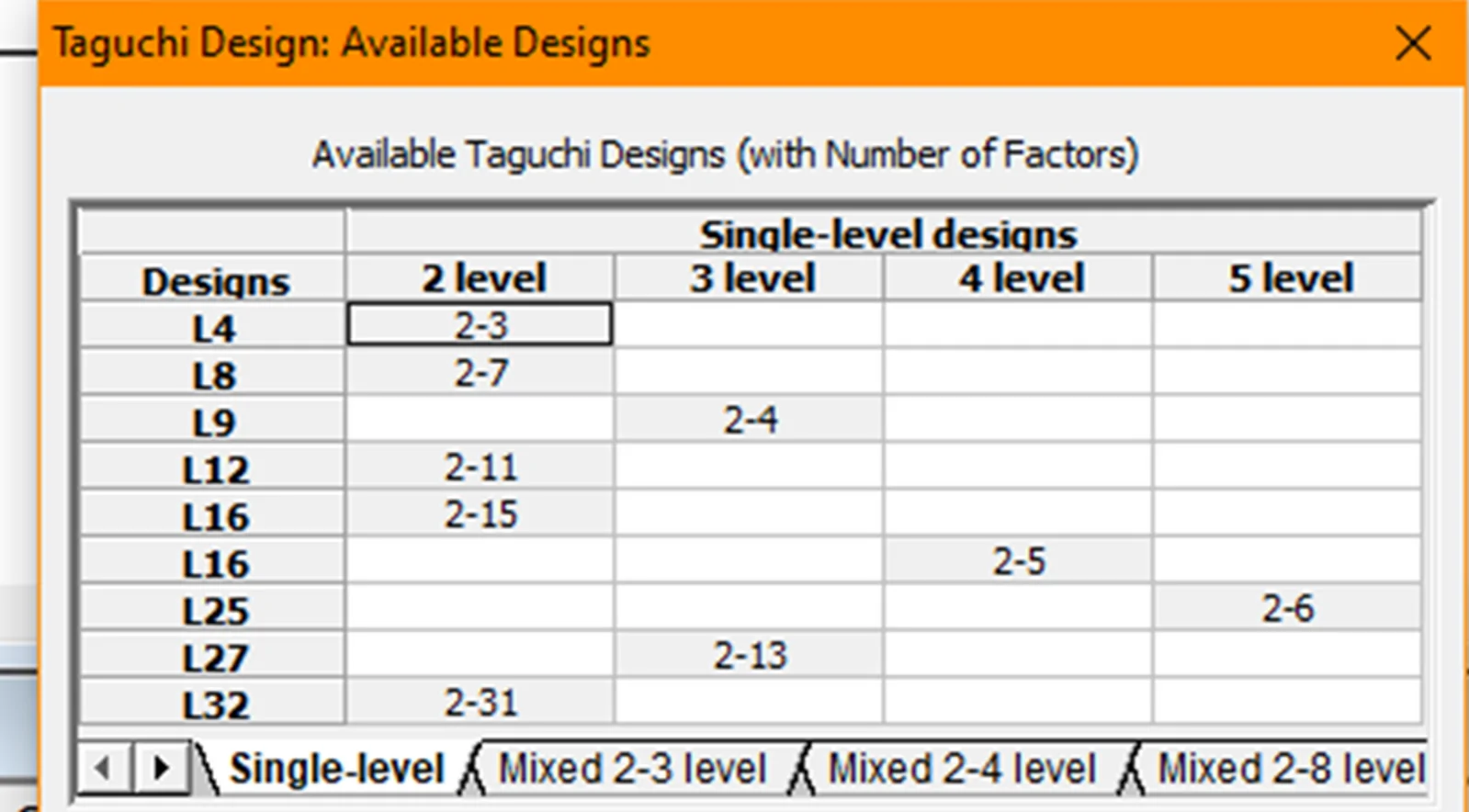
Taguchi L16 orthogonal array used for experimental design in the present study.

As indicated in Table 5, an orthogonal array was created after determining the control components, their levels, and responses. The Taguchi designs available to select orthogonal array is shown in Fig. 10. Taguchi technique is applied to conduct tests with an L_16_ (4 × 4) orthogonal array (16 tests: 4 variables and 4 levels) for untreated and cryogenically treated specimens.

### Experimentation

The material chosen for the present work is Al6061-SiC (13%)-Gr in varied proportions. Table 6 shows the experimental design for the current study, which uses an L16 orthogonal array. For wear analysis, many parameters such as reinforcement, load, speed, and time are considered. Control factors are defined as follows: A: Reinforcement (wt %), B: Speed (Rpm), C: Load (N), D: Time (Min). The experimental design was verified for consistency to ensure that each trial in the orthogonal array represents a unique combination of process parameters.

**Table 5:** Experimental control factors and their corresponding levels.

Control factors	Levels			
	I	II	III	IV
A: Reinforcement (wt%) SiC13 + Gr	0	1	2	3
B: Speed (rpm)	600	800	1000	1200
C: Load (N)	10	20	30	40
D: Time (Min)	5	10	15	20

* In MINITAB17 0, I, II, and III were used.

**Table 6:** Taguchi L16 orthogonal array used for experimental design.

Sl. No	Reinforcement (wt %)	Speed (rpm)	Load (*N*)	Time (min)
1.	0	600	10	5
2.	0	800	20	10
3.	0	1000	30	15
4.	0	1200	40	20
5.	1	600	20	15
6.	1	800	10	20
7.	1	1000	40	5
8.	1	1200	30	10
9.	2	600	30	20
10.	2	800	40	15
11.	2	1000	10	10
12.	2	1200	20	5
13.	3	600	40	10
14.	3	800	30	5
15.	3	1000	20	20
16.	3	1200	10	15

#### Pin-on-disc wear test

The wear behaviour of untreated and cryogenically treated hybrid Al6061-SiC-Gr composite specimens was evaluated using a DUCOM pin-on-disc tribometer under dry sliding conditions. Cylindrical wear specimens of 25 mm length and 10 mm diameter were tested against a hardened steel disc serving as the counter face. The wear test was conducted at room temperature under ambient laboratory conditions in air. A cantilever loading mechanism with a track radius of 100 mm was used to apply the normal load. During the test, wear scar depth and coefficient of friction were recorded for different combinations of reinforcement content, load, speed, and sliding time. Prior to testing, the specimens were ground using 200, 600, 800, and 1000 grit emery papers and cleaned with acetone to ensure a consistent test surface.

### Inference on the wear scar depth results obtained for the untreated specimens

The wear scar depth values obtained after experimenting are fed into the statistical software MINITAB 17 to know the most influencing parameters. Test conditions with output results of SN ratio for wear scar depth of untreated specimens using L_16_ orthogonal array are presented in Table 7.

#### Signal to noise (S/N) ratio analysis

The S/N ratio is calculated for the wear scar depth of the material as the response, considering the various elements. Equation 3.1 was used to get the S/N ratio.

Signal to Noise ratio (for smaller the better)

1 $$\: = 10\:\log \frac{1}{n}\sum (y)^{2}$$

where *n* = total number of readings and *y* = data collected.

**Fig. 11:**
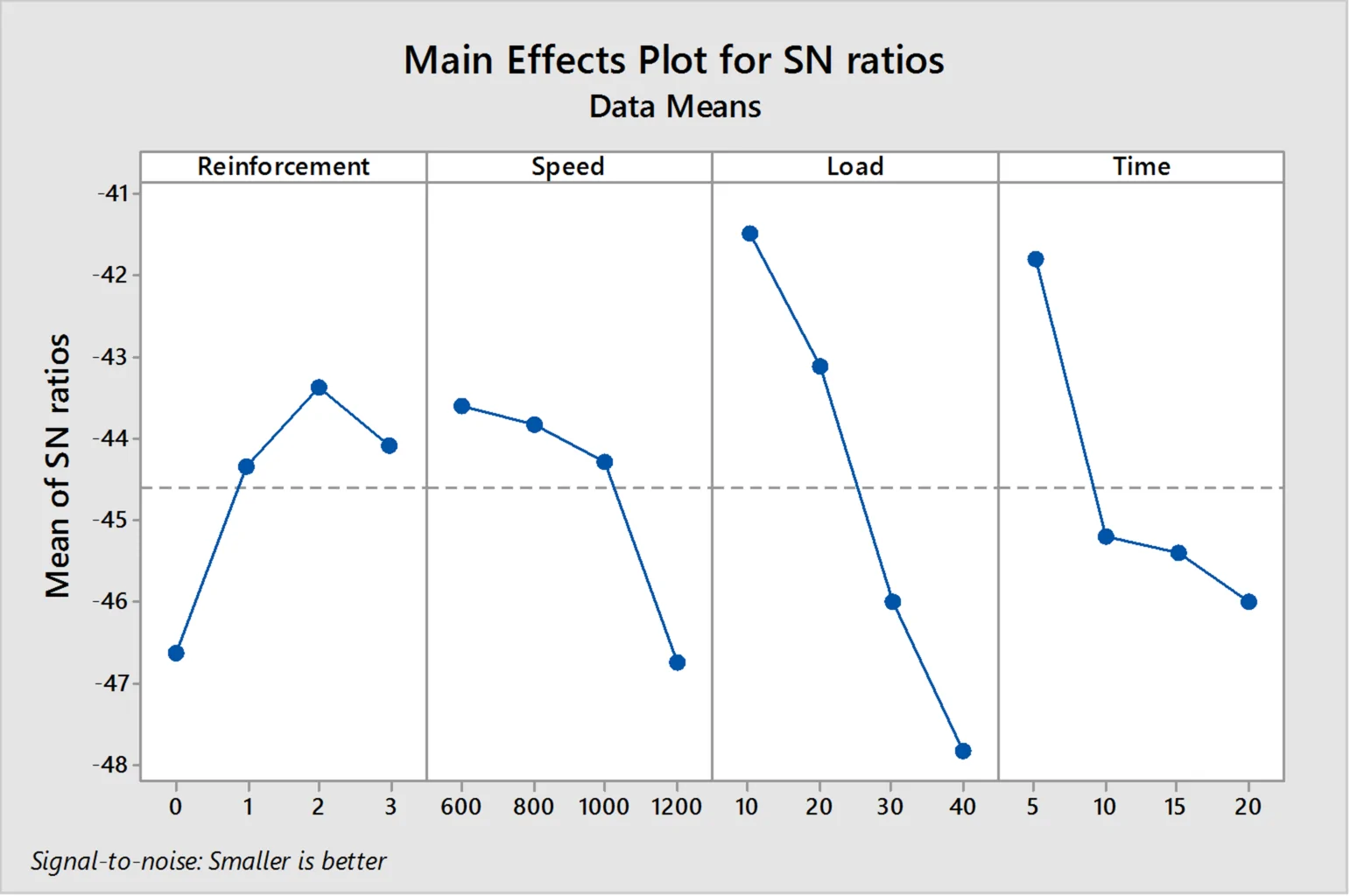
Main effects plot showing the influence of control factors (A: reinforcement, B: speed, C: load, D: time) on S/N ratio for untreated specimens.

The response values for untreated specimens’ S-N ratios (smaller is better) is shown in Table 8. The response and S/N ratios for each factor were plotted against each of its levels with a smaller-is-better condition for wear scar depth, as illustrated in Fig. 11.

Results show that the wear scar depth showed an increasing trend with varied values of reinforcement (A), load (B), speed (C), and time (D). The factors having a higher value of S/N ratio will satisfy the condition of minimum wear scar depth for optimal performance. Factors A, B, C, and D have a significant effect on wear scar depth. A combination of (A3-B1-C, and D1) gives minimum wear scar depth for specimens without cryogenic treatment. The signal-to-noise (S/N) ratio was calculated using the “smaller-the-better” criterion to evaluate wear performance.

**Table 7:** Wear scar depth and signal-to-noise (S/N) ratios for untreated specimens based on the Taguchi L16 orthogonal array.

Sl. No	A	B	C	D	Wear scar depth (µm)	S/*N* Ratio (dB)
1.	0	600	10	5	95	−39.5545
2.	0	800	20	10	180	−45.1055
3.	0	1000	30	15	300	−49.5424
4.	0	1200	40	20	410	−52.2557
5.	1	600	20	15	120	−41.5836
6.	1	800	10	20	140	−42.9226
7.	1	1000	40	5	170	−44.6090
8.	1	1200	30	10	260	−48.2995
9.	2	600	30	20	185	−45.3434
10.	2	800	40	15	210	−46.4444
11.	2	1000	10	10	94	−39.4626
12.	2	1200	20	5	130	−42.2789
13.	3	600	40	10	250	−47.9588
14.	3	800	30	5	110	−40.8279
15.	3	1000	20	20	150	−43.5218
16.	3	1200	10	15	160	−44.0824

#### Analysis of variance and regression

To evaluate the effect of individual parameters on wear scar depth, analysis of variance (ANOVA) was performed in conjunction with the Taguchi approach. ANOVA was used to quantify the percentage contribution of each parameter to the selected performance characteristic. The results, summarized in Table 9, were analysed at a 90% confidence level, with parameters exhibiting a p-value less than 0.1 considered statistically significant. The final column of Table 9 presents the percentage contribution of each parameter for specimens without cryogenic treatment. Among the factors, load exhibited the highest influence on wear scar depth (44.53%), followed by time (19.05%), reinforcement (18.73%), and speed (15.60%).

To further evaluate the predictive capability of the developed regression models, additional error metrics were considered. The root means square error (RMSE) and mean absolute error (MAE) were used to quantify the deviation between predicted and experimental values. The relatively low error values indicate reasonable agreement between model predictions and experimental observations, providing additional support for the adequacy of the developed models beyond the reported $\:{R}^{2}$values. Residual analysis was performed to assess model adequacy. The residuals were observed to be randomly distributed without any systematic pattern, suggesting that the regression models are appropriate and that the assumptions of linear regression are reasonably satisfied.

**Table 8:** S/N ratio response table for untreated specimens based on the smaller-is-better criterion.

Level	A (Reinforcement)	B (Speed)	C (Load)	D (Time)
I	−46.61	−43.61	−41.51	−41.82
II	−44.35	−43.83	−43.12	−45.21
III	−43.38	−44.28	−46	−45.41
IV	−44.1	−46.73	−47.82	−46.01
Delta	3.23	3.12	6.31	4.19
Rank	3	4	1	2

**Table 9:** Analysis of variance (ANOVA) for S/N ratios of untreated specimens based on adjusted sum of squares.

Source	DF	Adj SS	Adj MS	F-Value	*P*-value	Contribution (%)
Reinforcement	3	20,171	6723.5	8.88	0.053	18.73
Speed	3	16,793	5597.7	7.4	0.067	15.60
Load	3	47,956	15985.2	21.12	0.016	44.53
Time	3	20,515	6838.5	9.04	0.052	19.05
Residual error	3	2270	756.8			2.11
Total	15	107,705	6838.5			

To establish the correlation between the wear parameters namely reinforcement, load, speed, and time with the wear scar depth for untreated specimens’ linear regression model is used. The R-square value for the model summary is 97.90% which is well within the confidence limits. Separate regression models were developed for different reinforcement levels and treatment conditions (untreated and cryogenically treated specimens) to capture the variation in wear behaviour under distinct experimental settings. Although the functional form of the regression equations remains similar, differences in intercepts and coefficients reflect the influence of process parameters under varying material conditions.

2 $$WSD{\text{ }}in{\text{ }}micrometer{\text{ }} = {\text{ }}0.0{\text{ }} + ~0.0904 \times Speed{\text{ }} + ~4.430 \times Load{\text{ }} + ~4.95 \times Time$$

3 $$WSD{\text{ }}in{\text{ }}micrometer{\text{ }} = {\text{ }} - 81.5{\text{ }} + ~0.0904 \times Speed{\text{ }} + ~4.430 \times Load{\text{ }} + ~4.95 \times Time$$

4 $$WSD{\text{ }}in{\text{ }}micrometer{\text{ }} = {\text{ }} - 99.3{\text{ }} + ~0.0904 \times Speed{\text{ }} + ~4.430 \times Load{\text{ }} + ~4.95 \times Time$$

5 $$WSD{\text{ }}in{\text{ }}micrometer{\text{ }} = {\text{ }} - 86.5{\text{ }} + ~0.0904 \times Speed{\text{ }} + ~4.430 \times Load{\text{ }} + ~4.95 \times Time$$

Equations 2, 3, 4, and 5 gives the regression relation derived for wear scar depth (WSD) in micrometre of untreated specimens for reinforcement 0, 1, 2, and 3 respectively. The regression equations are derived by taking reinforcement as categorical predictors and load, time, and speed are taken as continuous predictors. The similarity in model structure arises from the use of a common regression framework, while variations in coefficients indicate differences in parameter sensitivity across reinforcement levels and treatment conditions.

#### Taguchi confirmation experiment

To confirm the accuracy of the analysis and validate the experimental results, the Taguchi confirmation test was used. Table 10 shows a comparison of the experimental and predicted values.

**Table 10:** Comparison of predicted and experimental results for untreated specimens.

Response	Optimal parameters (A3B1C1D1)	Predicted values (µm)	Experimental values (µm)	Error (µm)	Error (%)
Wear scar depth	A3B1C1D1	52.87	55.00	2.13	3.87

### Inference on the wear scar depth results obtained for the cryogenically treated specimens

The wear scar depth values obtained after experimenting are fed into the statistical software MINITAB 17 to know the most influencing parameters. Test conditions with output results of SN ratio for wear scar depth of cryogenically treated specimens using L_16_ orthogonal array are presented in Table 11. However, the absence of experimental replication limits the ability to estimate pure experimental error, and therefore the results should be interpreted with caution. Future studies may incorporate replicated experiments to further improve statistical robustness.

**Table 11:** Wear scar depth and S/N ratios for cryogenically treated specimens based on the Taguchi L16 orthogonal array.

Run	A	B	C	D	Wear scar depth (µm)	S/*N* ratio (dB)
1.	0	600	10	5	44	−32.8691
2.	0	800	20	10	85	−38.5884
3.	0	1000	30	15	142	−43.0458
4.	0	1200	40	20	190	−45.5751
5.	I	600	20	15	55	−34.8678
6.	I	800	10	20	64	−36.1555
7.	I	1000	40	5	80	−38.0618
8.	I	1200	30	10	120	−41.5836
9.	II	600	30	20	85	−38.5884
10.	II	800	40	15	95	−39.5545
11.	II	1000	10	10	45	−33.0643
12.	II	1200	20	5	61	−35.6590
13.	III	600	40	10	115	−41.2140
14.	III	800	30	5	48	−33.6248
15.	III	1000	20	20	70	−36.9020
16.	III	1200	10	15	78	−37.8419

#### Signal to noise (S/N) ratio analysis

**Fig. 12:**
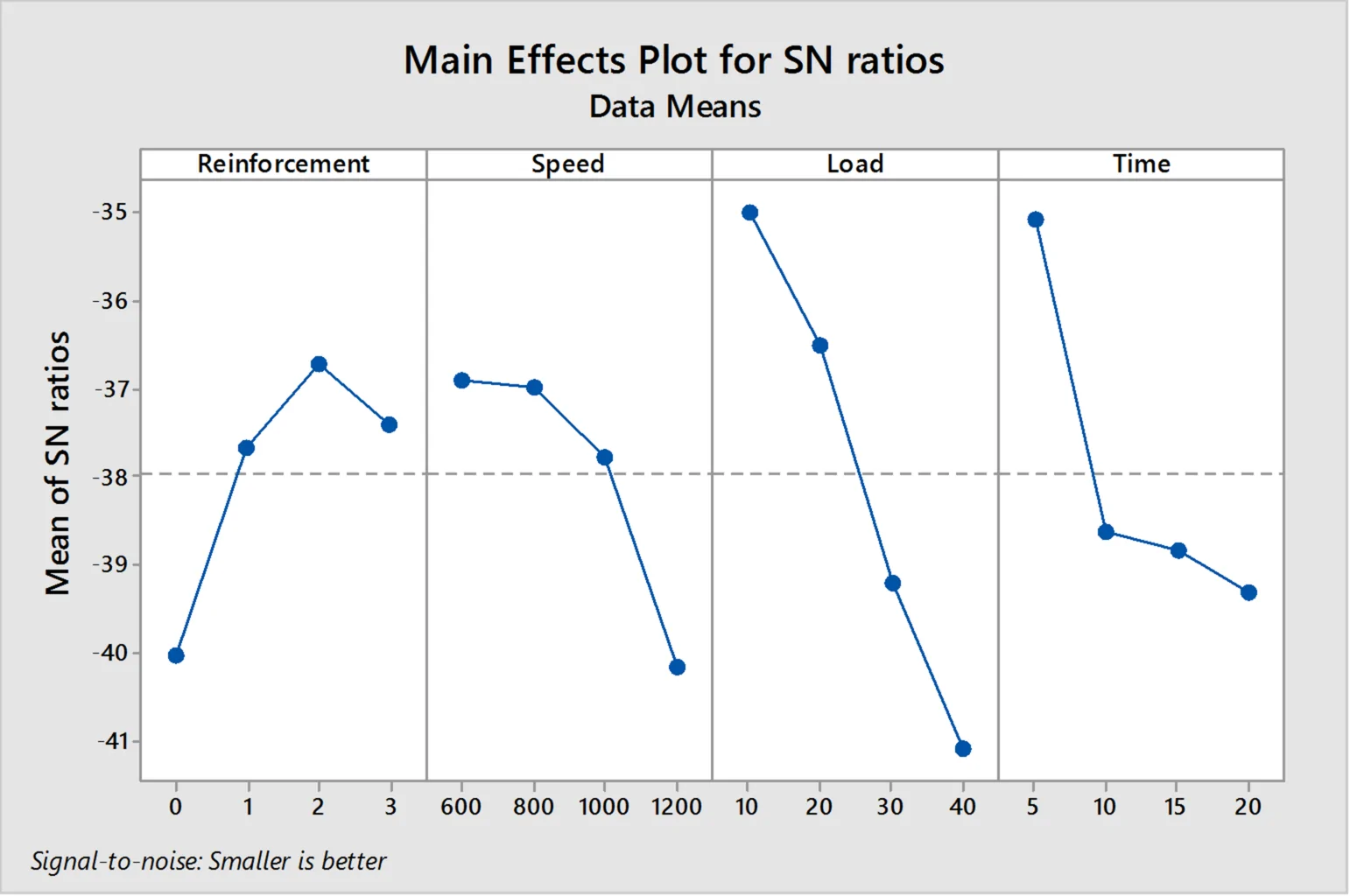
Main effects plot showing the influence of control factors (A: reinforcement, B: speed, C: load, D: time) on S/N ratio for cryogenically treated specimens.

The S/N ratio was calculated for the wear scar depth of the material as the response, considering the various elements. Using the equation in 3.1, the S/N ratio was computed. Table 12 shows the response table for S/N ratios (lower is preferable) of cryogenically treated specimens. The response and S/N ratios for each factor were plotted against each of its levels with a smaller-is-better condition for wear scar depth, as illustrated in Fig. 12.

**Table 12:** S/N ratio response table for cryogenically treated specimens based on the smaller-is-better criterion.

Level	A (Reinforcement)	B (Speed)	C (Load)	D (Time)
1	−40.02	−36.88	−34.98	−35.05
2	−37.67	−36.98	−36.5	−38.61
3	−36.72	−37.77	−39.21	−38.83
4	−37.4	−40.16	−41.1	−39.31
Delta	3.3	3.28	6.12	4.25
Rank	3	4	1	2

The wear scar depth was influenced by reinforcement, load, speed, and time. Higher S/N ratios correspond to conditions producing lower wear scar depth, indicating the significance of these factors in determining wear performance. A combination of A3, B1, C1, and D1 gives minimum wear scar depth for specimens with cryogenic treatment.

#### Analysis of variance and regression

Table 13 presents the ANOVA results evaluated at a 90% confidence level. Parameters with a *p*-value below 0.1 were considered to have a statistically significant effect on the performance metrics. From the Table 13 the final column gives the percentage contribution of each parameter for specimens subjected to cryogenic treatment. The load has the highest influence of 41.74% on wear scar depth followed by time, reinforcement, and speed. The time, reinforcement, and speed have an influence of 20.11%, 19.06%, and 16.79% respectively. The analysis of variance (ANOVA) was conducted at a 90% confidence level, which is commonly adopted in engineering design studies involving limited experimental datasets to balance statistical sensitivity and experimental constraints.

**Table 13:** Analysis of variance (ANOVA) for S/N ratios of cryogenically treated specimens based on adjusted sum of squares.

Source	DF	Adj SS	Adj MS	F-Value	*P*-Value	Contribution (%)
A (Reinforcement)	3	4446.9	1482.3	9.13	0.051	19.06
B (Speed)	3	3918.5	1306.2	8.04	0.06	16.79
C (Load)	3	9783.5	3261.2	20.08	0.017	41.74
D (Time)	3	4693.7	1564.6	9.63	0.048	20.11
Residual error	3	487.2	162.4			2.01
Total	15	23329.7				

To establish the correlation between the wear parameters namely reinforcement, load, speed, and time with the wear scar depth for cryogenically treated specimens’ linear regression model is used. The R-square value for the model summary is 97.90% which is well within the confidence limits.

6 $$WSD{\text{ }}in{\text{ }}micrometer{\text{ }} = {\text{ }}0.0{\text{ }} + ~0.0449 \times Speed{\text{ }} + ~1.990 \times Load{\text{ }} + ~2.305 \times Time$$

7 $$WSD{\text{ }}in{\text{ }}micrometer{\text{ }} = {\text{ }} - 39.1{\text{ }} + ~0.0449 \times Speed{\text{ }} + ~1.990 \times Load{\text{ }} + ~2.305 \times Time$$

8 $$WSD{\text{ }}in{\text{ }}micrometer{\text{ }} = {\text{ }} - 47.5{\text{ }} + ~0.0449 \times Speed{\text{ }} + ~1.990 \times Load{\text{ }} + ~2.305 \times Time$$

9 $$WSD{\text{ }}in{\text{ }}micrometer{\text{ }} = {\text{ }} - 41.2{\text{ }} + ~0.0449 \times Speed{\text{ }} + ~1.990 \times Load{\text{ }} + ~2.305 \times Time$$

Equations 6, 7, 8, and 9 gives the regression relation derived for wear scar depth in micrometer of cryogenically treated specimens for reinforcement 0, 1, 2, and 3 respectively. The regression equations are derived by taking reinforcement as categorical predictors and load, time, and speed are taken as continuous predictors.

**Table 14:** Model validation results showing comparison of predicted and experimental values for wear scar depth and coefficient of friction.

Response	Condition	*R*^2^ (%)	RMSE	MAE
Wear scar depth	Untreated	97.90	2.13	2.13
Wear scar depth	Cryogenically treated	97.90	3.26	3.26
Coefficient of friction	Untreated	95.50	0.0013	0.0013
Coefficient of friction	Cryogenically treated	92.30	0.0006	0.0006

RMSE and MAE values are expressed in the same units as the respective response variables (µm for wear scar depth and dimensionless for coefficient of friction).

The predictive performance of the developed regression models was evaluated using the coefficient of determination (R²), root mean square error (RMSE), and mean absolute error (MAE), as presented in Table 14. The high R² values and relatively low RMSE and MAE values indicate good agreement between predicted and experimental results, supporting the adequacy of the developed models. However, the validation is based on limited confirmation experiments, and the absence of experimental replication restricts the estimation of pure experimental error and statistical variability. Consequently, uncertainty in the present study is inferred through model validation metrics and residual analysis rather than direct estimation of confidence intervals. The residuals were observed to be randomly distributed, suggesting that the regression models are appropriate within the scope of the experimental design. Nevertheless, the results should be interpreted with appropriate caution due to the limitations of the Taguchi design, which does not include replication. Future studies incorporating replicated experiments and statistical dispersion measures are recommended to further quantify variability and improve model robustness. The observed trends are based on discrete experimental levels, and no assumption of strict linearity is made.

#### Taguchi confirmation experiment

The Taguchi confirmation test was employed to validate the experimental results and verify the accuracy of the analysis. Table 15 shows the comparison between the expected and actual value.

**Table 15:** Comparison of predicted and experimental results for cryogenically treated specimens (confirmation experiment).

Response	Optimal parameters (A3B1C1D1)	Predicted values (µm)	Experimental values (µm)	Error (µm)	Error (%)
Wear scar depth	A3B1C1D1	24.74	28	3.26	11.64

The confirmation experiment results show reasonable agreement between predicted and experimental values, with a deviation of approximately 11.64%, indicating acceptable predictive capability of the developed model for cryogenically treated specimens.

### Inference on the coefficient of friction results obtained for the untreated specimens

The coefficient of friction values obtained after experimenting are fed into the statistical software MINITAB 17 to know the most influencing parameters. Test conditions with output results of SN ratio for the coefficient of friction of untreated specimens using L_16_ orthogonal array are presented in Table 16. The results indicate that the coefficient of friction varies with process parameters, with lower values observed under optimized conditions.

**Table 16:** Coefficient of friction and S/N ratios for untreated specimens based on the Taguchi L16 orthogonal array.

Run	A	B	C	D	Coefficient of friction	S/*N* ratio (dB)
1.	0	600	10	5	0.051000	25.8486
2.	0	800	20	10	0.099000	20.0873
3.	0	1000	30	15	0.158000	16.0269
4.	0	1200	40	20	0.231000	12.7278
5.	I	600	20	15	0.066000	23.6091
6.	I	800	10	20	0.074000	22.6154
7.	I	1000	40	5	0.096000	20.3546
8.	I	1200	30	10	0.139000	17.1397
9.	II	600	30	20	0.096000	20.3546
10.	II	800	40	15	0.107000	19.4123
11.	II	1000	10	10	0.045000	26.9357
12.	II	1200	20	5	0.066000	23.6091
13.	III	600	40	10	0.111429	19.0601
14.	III	800	30	5	0.053000	25.5145
15.	III	1000	20	20	0.086000	21.3100
16.	III	1200	10	15	0.096000	20.3546

#### Signal to noise (S-N) ratio analysis

**Fig. 13:**
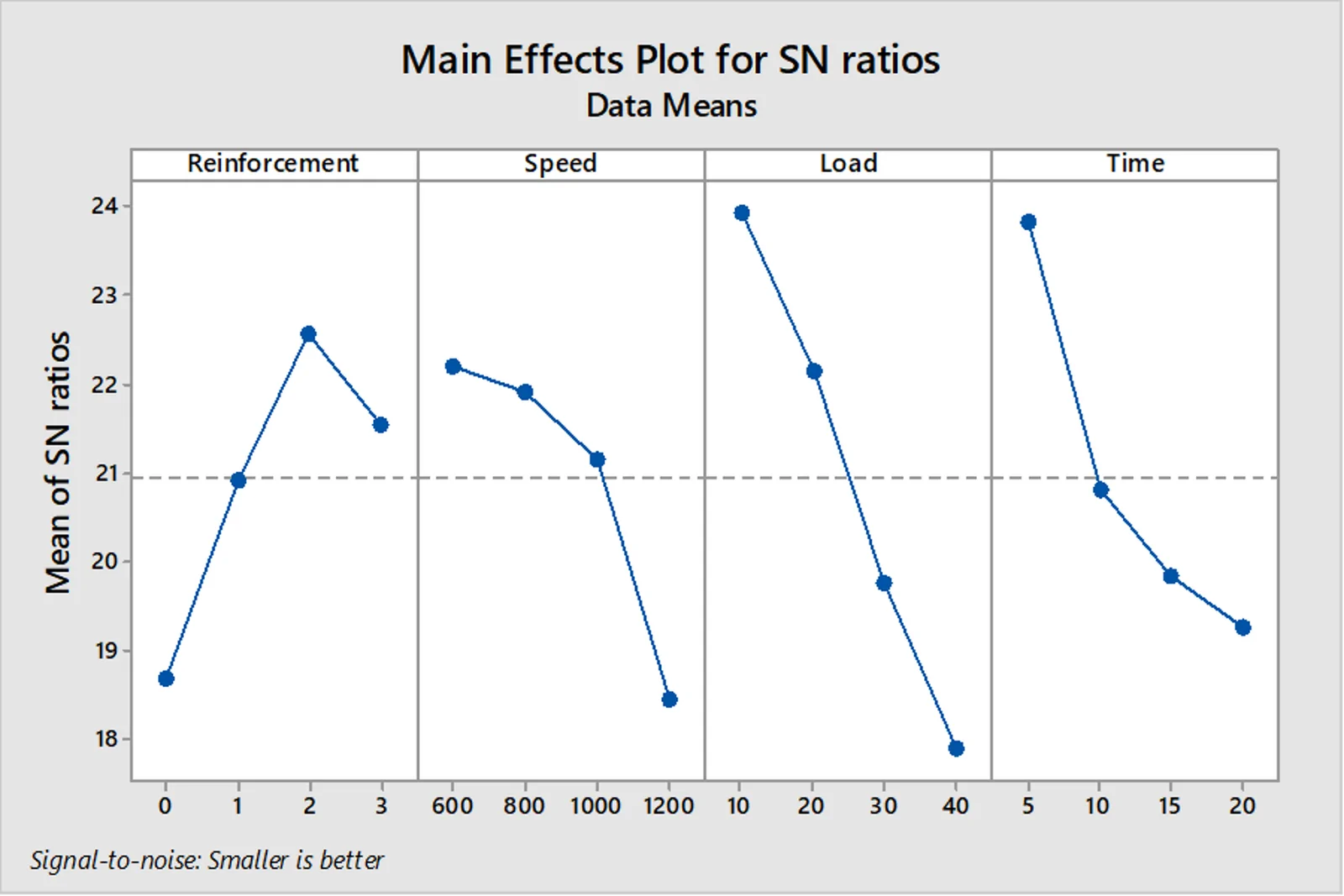
Taguchi Main Effects Plot of S/N Ratios (Untreated Specimens).

Equation 1 was utilized to determine the coefficient of friction’s signal-to-noise (S/N), considering the influence of different parameters. Table 17 presents the response table for untreated specimens, evaluated under the ‘smaller-is-better’ criterion. Figure 13 shows the plot of response and S/N ratios for each factor. The results indicate that the coefficient of friction showed an increasing trend with changes in reinforcement (A), load (B), speed (C), and time (D). Factors with higher S/N ratio values corresponded to lower coefficients of friction, ensuring optimal performance. All four factors (A, B, C, and D) exhibited significant effects, with the optimal parameter combination identified as A3–B1–C1–D1 for untreated specimens.

**Table 17:** S/N ratio response table for coefficient of friction of untreated specimens based on the smaller-is-better criterion.

Level	A (Reinforcement)	B (Speed)	C (Load)	D (Time)
1	18.67	22.22	23.94	23.83
2	20.93	21.91	22.15	20.81
3	22.58	21.16	19.76	19.85
4	21.56	18.46	17.89	19.25
Delta	3.91	3.76	6.05	4.58
Rank	3	4	1	2

#### Analysis of variance and regression

Table 18 presents the ANOVA results evaluated at a 90% confidence level, with parameters showing a *p* ≤ 0.1 which will be considered statistically significant. The final column reports the percentage contribution of each parameter for untreated specimens. Among the factors, load exhibited the greatest influence on the coefficient of friction (39.98%), followed by time (23.45%), reinforcement (16.58%), and speed (15.51%).

To establish the correlation between the wear parameters namely reinforcement, load, speed, and time with the coefficient of friction for untreated specimens’ linear regression model is used. The R-square value for the model summary is 95.50% which is well within the confidence limits.

10 $$COF{\text{ }} = {\text{ }} - 0.0451{\text{ }} + ~0.000084 \times Speed{\text{ }} + ~0.002418 \times Load{\text{ }} + ~0.003478 \times Time$$

11 $$COF{\text{ }} = {\text{ }} - 0.0861{\text{ }} + ~0.000084 \times Speed{\text{ }} + ~0.002418 \times Load{\text{ }} + ~0.003478 \times Time$$

12 $$COF{\text{ }} = {\text{ }} - 0.1013{\text{ }} + ~0.000084 \times Speed{\text{ }} + ~0.002418 \times Load{\text{ }} + ~0.003478 \times Time$$

13 $$COF{\text{ }} = {\text{ }} - 0.0932{\text{ }} + ~0.000084 \times Speed{\text{ }} + ~0.002418 \times Load{\text{ }} + ~0.003478 \times Time$$

Equations 10, 11, 12, and 13 gives the regression relation derived for the Coefficient of Friction (COF) of untreated specimens for reinforcement 0, 1, 2, and 3 respectively. The regression equations are derived by taking reinforcement as categorical predictors and load, time, and speed are taken as continuous predictors.

**Table 18:** Analysis of variance (ANOVA) for S/N ratios of coefficient of friction for untreated specimens based on adjusted sum of squares.

Source	DF	Adj SS	Adj MS	F-Value	*P*-Value	Contribution %
Reinforcement	3	35.110	11.703	3.69	0.156	16.58
Speed	3	32.832	10.944	3.45	0.168	15.51
Load	3	84.682	28.227	8.90	0.053	39.98
Time	3	49.664	16.555	5.22	0.104	23.45
Residual error	3	9.519	3.173			4.45
Total	15	211.806				

#### Taguchi confirmation experiment

The Taguchi confirmation test was performed to validate the experimental results and verify the accuracy of the analysis. Table 19 presents a comparison between the predicted and actual values. The confirmation experiment results show reasonable agreement between predicted and experimental values, with a deviation of approximately 10.00%, indicating acceptable predictive capability of the developed model.

**Table 19:** Comparison of predicted and experimental results for untreated specimens (confirmation experiment).

Response	Optimal parameters (A3B1C1D1)	Predicted values (µm)	Experimental values (µm)	Error (µm)	Error (%)
Wear scar depth	A3B1C1D1	0.0117	0.0130	0.0013	10.00

### Inference on the coefficient of friction results obtained for the cryogenically treated specimens

The experimental wear scar depth values were analysed using MINITAB 17 to identify the most influential parameters. Test conditions with output results of SN ratio for the coefficient of friction of cryogenically treated specimens using L_16_ orthogonal array are presented in Table 20.

**Table 20:** Coefficient of friction and S/N ratios for cryogenically treated specimens based on the Taguchi L16 orthogonal array.

Run	A	B	C	D	Coefficient of friction	S/*N* ratio (dB)
1.	0	600	10	5	0.0260	31.7005
2.	0	800	20	10	0.0505	25.9392
3.	0	1000	30	15	0.0800	21.9382
4.	0	1200	40	20	0.0963	20.3320
5.	I	600	20	15	0.0380	28.4043
6.	I	800	10	20	0.0370	28.6360
7.	I	1000	40	5	0.0535	25.4352
8.	I	1200	30	10	0.0734	22.6907
9.	II	600	30	20	0.0470	26.5580
10.	II	800	40	15	0.0580	26.6314
11.	II	1000	10	10	0.0230	32.7654
12.	II	1200	20	5	0.0400	27.9588
13.	III	600	40	10	0.0700	23.0980
14.	III	800	30	5	0.0300	30.4702
15.	III	1000	20	20	0.0500	26.0143
16.	III	1200	10	15	0.0430	27.3306

#### Signal to noise (S/N) ratio analysis

**Fig. 14:**
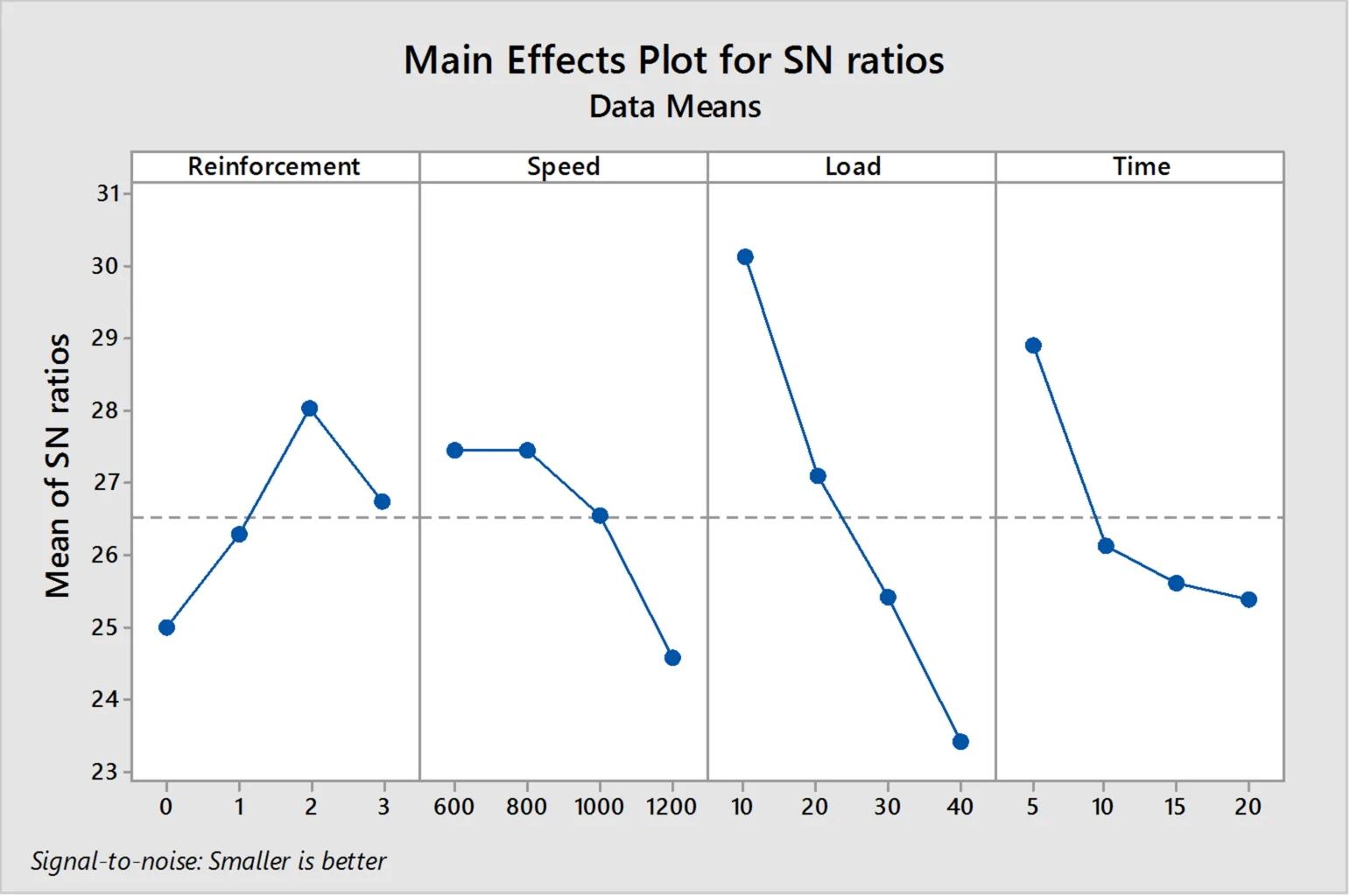
Main effects plot showing the influence of control factors (A: reinforcement, B: speed, C: load, D: time) on S/N ratio for coefficient of friction of cryogenically treated specimens.

The S/N ratio was calculated for the coefficient of friction of the material as the reaction, considering the various elements. Using the equation in 1, the S/N ratio was computed. Table 21 shows the response table for untreated specimens’ S/N ratios (lower is preferable). The response and S/N ratios for each factor were plotted against each of its levels with a smaller-is-better condition for the coefficient of friction, as illustrated in Fig. 14.

The results indicated that wear scar depth showed an increasing trend with changes in reinforcement (A), load (B), speed (C), and time (D). Factors with higher S/N ratios corresponded to lower coefficients of friction, meeting the criterion for optimal performance. All four factors (A, B, C, and D) had a significant influence on the coefficient of friction, with the optimal parameter combination for untreated specimens identified as A3–B1–C1–D1. The delta values indicate that load is the most influential parameter affecting the coefficient of friction for cryogenically treated specimens.

**Table 21:** S/N ratio response table for coefficient of friction of cryogenically treated specimens based on the smaller-is-better criterion.

Level	A (Reinforcement)	B (Speed)	C (Load)	D (Time)
1	24.98	27.44	30.11	28.89
2	26.29	27.44	27.08	26.12
3	28.00	26.54	25.41	25.60
4	26.73	24.58	23.40	25.39
Delta	3.03	2.87	6.71	3.51
Rank	3	4	1	2

#### Analysis of variance and regression

Table 22 presents the ANOVA results evaluated at a 90% confidence level, where parameters with a *p* ≤ 0.1 were which will be considered statistically significant. The final column summarizes the percentage contribution of each parameter for cryogenically treated specimens. Among the factors, load exhibited the greatest influence on the coefficient of friction (52.83%), followed by time (17.31%), reinforcement (10.22%), and speed (3.99%).

**Table 22:** Analysis of variance (ANOVA) for S/N ratios of coefficient of friction for cryogenically treated specimens based on adjusted sum of squares.

Source	DF	Adj SS	Adj MS	F-Value	*P*-Value	Contribution (%)
A (Reinforcement)	3	18.70	6.232	1.33	0.409	10.22
B (Speed)	3	7.295	1.56	1.56	0.361	3.99
C (Load)	3	96.59	32.197	6.89	0.074	52.83
D (Time)	3	31.64	10.548	2.26	0.260	17.31
Residual Error	3	14.01	14.01	4.670		7.66
Total	15	182.82				

To establish the correlation between the wear parameters namely reinforcement, load, speed, and time with the coefficient of friction for untreated specimens’ linear regression model is used. The R-square value for the model summary is 92.3% which is well within the confidence limits.

14 $$COF{\text{ }} = {\text{ }} - 0.0109{\text{ }} + ~0.000031 \times Speed{\text{ }} + ~0.001245 \times Load{\text{ }} + ~0.001224 \times Time$$

15 $$COF{\text{ }} = {\text{ }} - 0.0236{\text{ }} + ~0.000031 \times Speed{\text{ }} + ~0.001245 \times Load{\text{ }} + ~0.001224 \times Time$$

16 $$COF{\text{ }} = {\text{ }} - 0.0321{\text{ }} + ~0.000031 \times Speed{\text{ }} + ~0.001245 \times Load{\text{ }} + ~0.001224 \times Time$$

17 $$COF{\text{ }} = {\text{ }} - 0.0258{\text{ }} + ~0.000031 \times Speed{\text{ }} + ~0.001245 \times Load{\text{ }} + ~0.001224 \times Time$$

Equations 15, 16, 17, and 18 gives the regression relation derived for the Coefficient of Friction (COF) of cryogenically treated specimens for reinforcement 0, 1, 2, and 3 respectively. The regression equations are derived by taking reinforcement as categorical predictors and load, time, and speed are taken as continuous predictors.

#### Taguchi confirmation experiment

The Taguchi confirmation test was conducted to validate the experimental findings and verify the accuracy of the analysis. Table 23 provides a comparison between the predicted and actual values. The confirmation experiment results show good agreement between predicted and experimental values, with a deviation of approximately 1.50%, indicating strong predictive capability of the developed model for cryogenically treated specimens.

**Table 23:** Comparison of predicted and experimental results for cryogenically treated specimens (confirmation experiment).

Response	Optimal parameters (A3B1C1D1)	Predicted values (µm)	Experimental values (µm)	Error (µm)	Error (%)
Wear scar depth	A3B1C1D1	0.00395	0.00400	0.00006	1.50

### Summary

From Sect. 3.3 to 3.6, factors A, B, C, and D were found to significantly influence both wear scar depth and coefficient of friction. The optimal parameter combination (A3–B1–C1–D1) yielded the lowest values for both untreated and cryogenically treated specimens. Among the parameters, load exhibited the greatest influence, followed by time, reinforcement, and speed. Furthermore, under the same optimal combination (A3–B1–C1–D1), cryogenically treated specimens demonstrated lower wear scar depth and coefficient of friction compared to untreated specimens (see Table 24).

**Table 24:** Comparison of wear scar depth and coefficient of friction for untreated and cryogenically treated specimens at optimal conditions (A3B1C1D1).

Response	Condition	Value
Wear scar depth (µm)	Untreated	55.00
Wear scar depth (µm)	Cryogenically treated	28.00
Coefficient of friction	Untreated	0.0130
Coefficient of friction	Cryogenically treated	0.0040

**Table 25:** Comparative analysis of wear scar depth and coefficient of friction for untreated and cryogenically treated specimens at optimal conditions (A3B1C1D1), with percentage reduction achieved through cryogenic treatment.

Response	Untreated	Cryogenically treated	Improvement (%)
Wear scar depth (µm)	55.00	28.00	49.09
Coefficient of friction	0.0130	0.0040	69.23

The cryogenic treatment resulted in a reduction of approximately 49.09% in wear scar depth and 69.23% in coefficient of friction compared to untreated specimens under optimal conditions (Table 25).

### Worn surface morphology

According to ASTM standards, wear tests on aluminum MMC can be performed under sliding conditions utilizing pin-on-disc testing apparatus, typically at room temperature and without lubrication. To maintain proper contact with the rotating disc, the test pins are machined and polished prior to testing. During the experiment, controlled loads are applied to press the specimen pins against a hardened steel counter face. The tests are conducted by varying operational parameters such as applied load, sliding velocity, sliding distance, and reinforcement content. Wear performance is primarily evaluated in terms of wear scar depth and coefficient of friction. The relative sliding motion between surfaces leads to surface degradation, although the use of solid lubricants can substantially reduce this effect^35^. Accordingly, this section presents the analysis of worn surface morphologies of Al/SiC/Gr hybrid composite specimens.

The tribological behaviour of the Al6061–SiC–Gr hybrid composites can be understood based on the distinct functional roles of the reinforcement phases. Silicon carbide (SiC) acts as a hard load-bearing reinforcement within the aluminium matrix. Due to its high hardness and stiffness, SiC particles restrict plastic deformation of the matrix and improve the load-carrying capacity during sliding, thereby reducing the severity of ploughing and material removal. This results in a decrease in wear scar depth, particularly at higher reinforcement levels. In contrast, graphite functions as a solid lubricant. During sliding, graphite particles tend to smear across the contact interface and form a lubricious tribofilm, which reduces direct metal-to-metal contact and lowers interfacial shear strength. This mechanism contributes to a reduction in the coefficient of friction and promotes smoother wear tracks. The combined presence of SiC and graphite produces a synergistic effect, where SiC enhances wear resistance through load support while graphite minimizes friction through tribofilm formation, as reported in recent tribological studies^36^. These findings are consistent with previous studies showing that hybrid reinforcement systems combining hard ceramic particles and solid lubricants simultaneously improve wear resistance and reduce friction. The ceramic phase enhances hardness and load-bearing capacity, whereas the carbon-based phase promotes lubricating film formation at the sliding interface, thereby reducing shear stress and adhesive wear. These observations align with recent studies on multi-reinforcement aluminium matrix composites, where synergistic interactions between ceramic and carbonaceous reinforcements significantly enhance tribological performance^37^.

The observed worn surface features correlate well with the measured wear scar depth and coefficient of friction. Deeper grooves and severe surface damage indicate increased material removal and higher wear, whereas smoother surfaces with shallow grooves reflect improved wear resistance. Similarly, rougher surfaces tend to increase friction due to greater asperity interaction, while smoother surfaces promote more stable sliding conditions. Studies on surface topography have shown that variations in roughness parameters significantly affect friction behaviour and wear evolution, with increased roughness generally leading to more severe wear and deformation patterns.

**Fig. 15:**
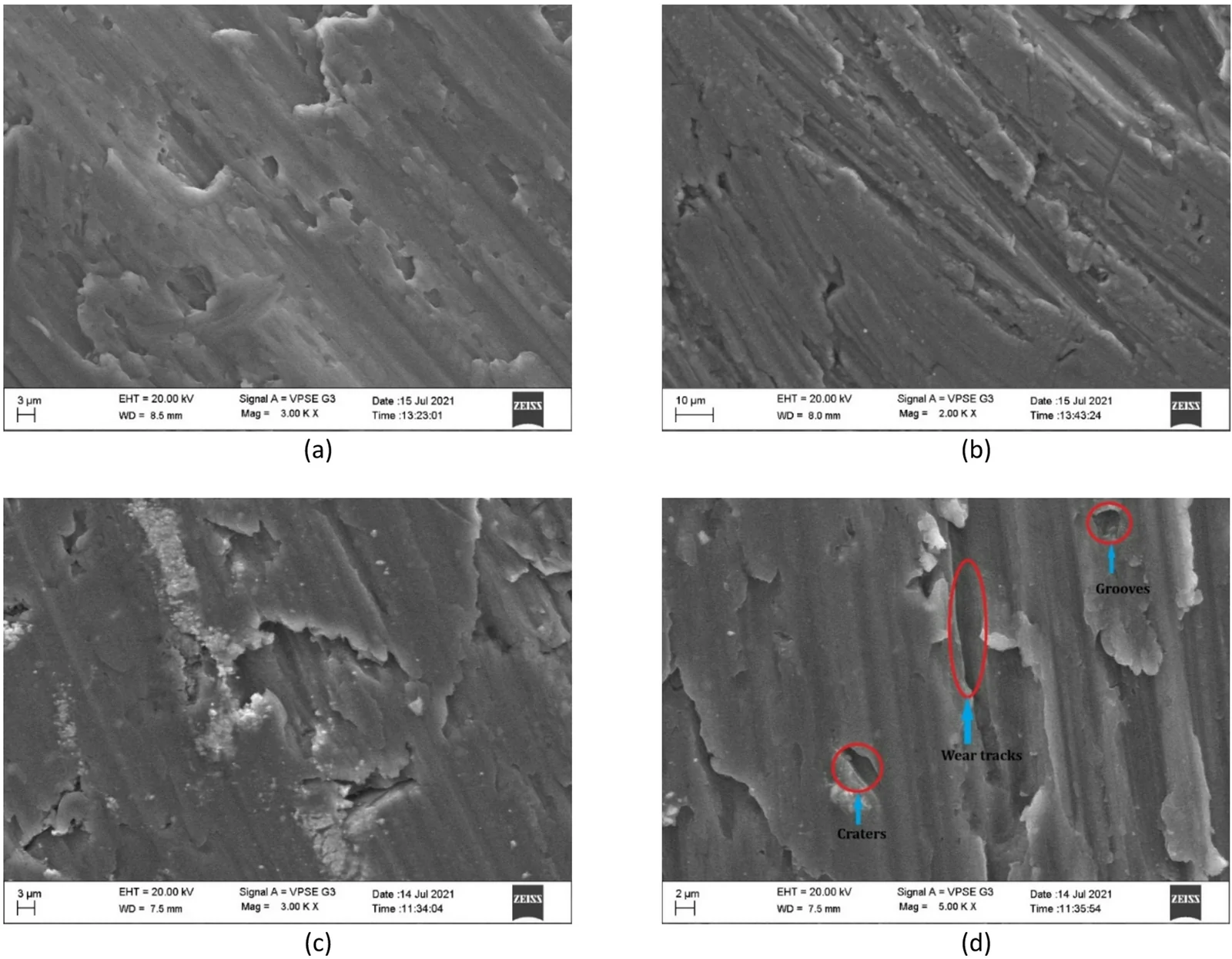
SEM micrographs of worn surfaces of untreated specimens under a maximum load of 40 N: (**a**) Al6061 base alloy, (**b**) Al86SiC13Gr1, (**c**) Al85SiC13Gr2, and (**d**) Al84SiC13Gr3 (magnification: 1000×; scale bar: 50 μm).

**Fig. 16:**
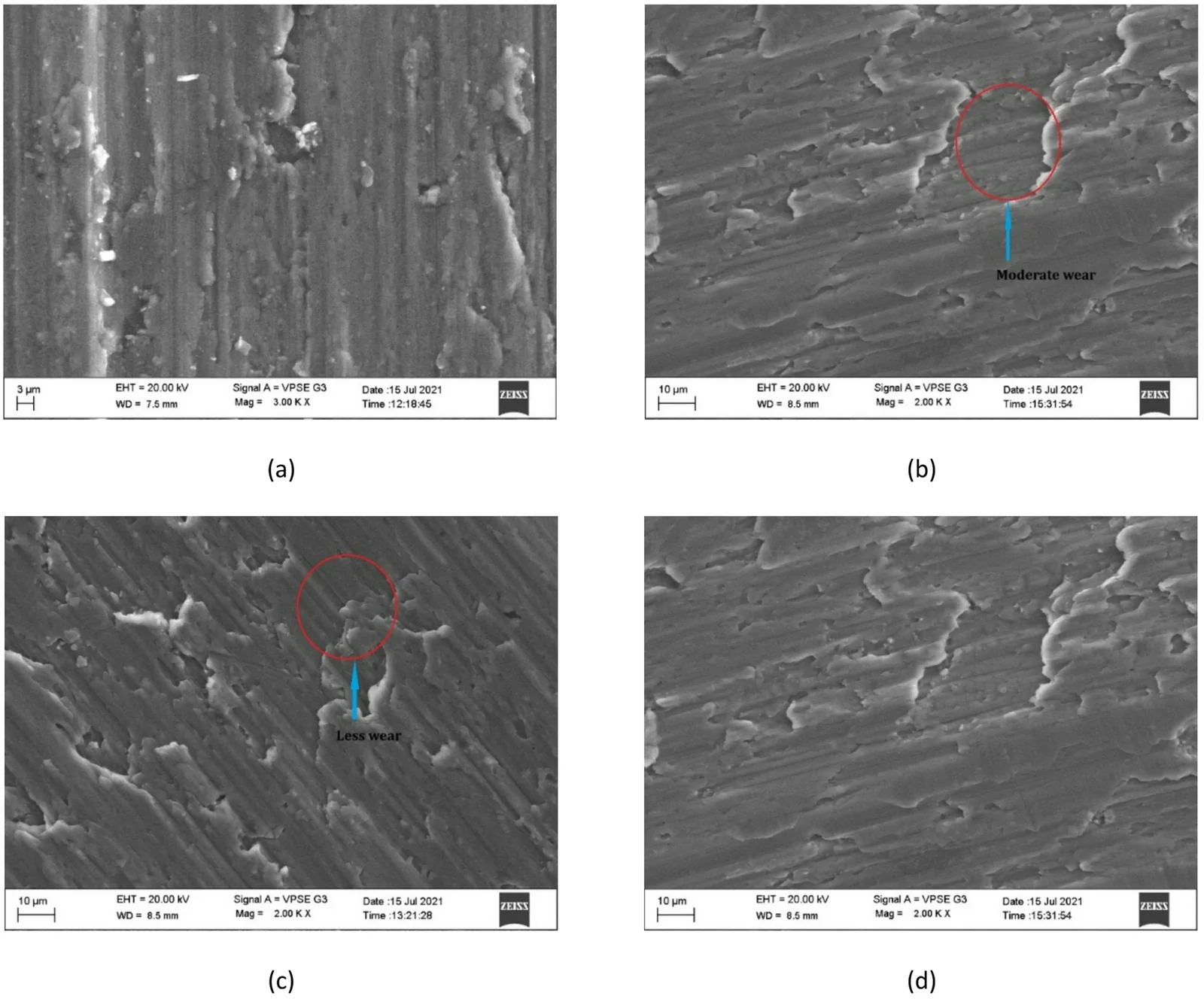
SEM micrographs of worn surfaces of cryogenically treated specimens: (**a**) Al6061 base alloy, (**b**) Al86SiC13Gr1-CT, (**c**) Al85SiC13Gr2-CT, and (**d**) Al84SiC13Gr3-CT at a maximum load of 40 N (magnification: 1000×; scale bar: 50 μm).

The study of worn surfaces on untreated and cryogenically treated as-cast and composite specimens is depicted in Figs. 15 and 16. Figure 15 a-d shows Scanning Electron Microscope images of worn surfaces of untreated as-cast and composite specimens. Recrystallized grains, SiC particle dispersion in the matrix’s interdendritic regions, clustered areas of particulates and pores compose the microstructure. On worn surfaces, fractured regions and interfacial cracks are detected. These surface features indicate localized stress concentration and material removal, which directly contribute to increased wear scar depth under higher loading conditions. The surface of Fig. 15 a exhibits smaller grooves and slight deformation at the groove margins. Due to a lower proportion of reinforcements and higher loads, the plastic deformation on the composite surface seems to be severe, as seen in Fig. 15 b. Wear resistance is primarily influenced by the coarse particle content of the composite, and resistance rises as SiC particle concentration increases. Due to the extensive penetration of the SiC particles, longitudinal wear tracks can be seen and because of the hard nature of SiC particles, distorted patches can be noticed on the worn track surfaces^38^. Figure 15 c shows that the wear scar depth of hybrid composites reduces as reinforcement content increases, because of significant quantity of SiC and Gr particles^39^. The very fine surface scratches develop to distinct grooves as the load increases, and damaged patches in the shape of craters have been seen in the places where particles are pushed out as shown in Fig. 15 d. The development of grooves by ploughing of hard asperities of the counter face characterizes the wear processes, and temperature rise is one of the most essential aspects^40^. Because of the high filler content and lubricant nature of Graphite, the longitudinal wear tracks seem to be parallel and homogeneous as seen in Fig. 15 d. Because of the graphite reinforcing, the surfaces also seemed to be smooth. This smoother surface condition is associated with lower friction values, as reduced asperity interaction and the formation of a lubricating layer facilitate easier sliding at the interface. This smoother surface morphology is consistent with the formation of a graphite-rich tribofilm at the sliding interface, which reduces adhesive interaction and stabilizes the wear process. Although surface topography-wear relationships are inferred from morphological observations and tribological data, detailed surface roughness quantification was not performed in the present study.

The morphological features observed in the SEM micrographs can be related to the measured wear responses in a semi-quantitative manner. Specimens exhibiting deeper and wider grooves and extensive plastic deformation correspond to higher wear scar depth, indicating dominant abrasive wear. In contrast, shallower grooves, smoother tracks, and reduced debris accumulation are associated with lower wear scar depth. Similarly, surfaces showing pronounced asperity interaction and fragmented debris correspond to higher coefficients of friction, whereas more uniform surfaces with reduced asperity interaction are associated with lower friction. These observations are consistent with a transition from severe abrasive/adhesive wear in untreated specimens to a more stable wear regime in cryogenically treated composites.

The coefficient of friction is also strongly influenced by surface topography, where rougher surfaces with prominent asperities increase interfacial shear resistance, while smoother surfaces promote stable sliding and reduced friction. Such relationships between surface topography, friction, and wear behaviour have been widely reported in recent studies, highlighting the critical role of surface roughness and asperity interaction in tribological performance^41,42^.

SEM micrographs of cryogenically treated as-cast and composite surfaces are presented in Fig. 16a–d. The worn surface of the base alloy (Fig. 16a) exhibits relatively smoother regions with mild grooves and cavities, indicating limited resistance to deformation under sliding conditions. In contrast, the reinforced composites display distinct wear features governed by the presence of SiC and graphite particles. The composite shown in Fig. 16b exhibits moderate wear characterized by shallow grooves and localized deformation. With increasing reinforcement content, as observed in Fig. 16c, the wear scar depth is reduced, which can be attributed to improved load-bearing capacity and resistance to plastic deformation. The cryogenically treated specimen in Fig. 16d exhibits more uniform and shallower wear tracks, with grooves aligned along the sliding direction due to the interaction of hard reinforcement particles with the counterface. The presence of dimples suggests localized material removal and micro-ploughing mechanisms. The observed reduction in groove depth and smoother surface morphology in cryogenically treated composites correspond well with the experimentally measured lower wear scar depth and coefficient of friction, indicating enhanced resistance to both abrasive and adhesive wear mechanisms. These results support the improved tribological performance of cryogenically treated hybrid composites compared to untreated specimens. Although tribofilm formation and interfacial effects are inferred from the observed wear behaviour and surface morphology, direct chemical and interfacial characterization was not performed in the present study.

The observed tribological behaviour of the developed composites can be understood through a process-structure-property-performance relationship. The selected process parameters optimized using the Taguchi approach, along with cryogenic treatment, influence the microstructural characteristics of the composite, including reinforcement distribution, matrix refinement, and interfacial stability. These structural modifications are reflected in the worn surface morphology observed through SEM, where cryogenically treated specimens exhibit smoother surfaces, reduced grove depth, and limited plastic deformation compared to untreated specimens. These microstructural and surface characteristics directly influence the measured properties, namely wear scar depth and coefficient of friction. Reduced groove formation and smoother wear tracks correspond to lower wear scar depth, while decreased asperity interaction and improved surface stability contribute to reduced friction. The presence of a smoother and more continuous surface layer is consistent with reduced interfacial shear and supports the observed decrease in coefficient of friction.

Consequently, the improved tribological performance of cryogenically treated Al6061-SiC-Gr composites can be attributed to the combined effect of optimized processing conditions and favourable microstructural evolution. This integrated interpretation provides a comprehensive understanding of how process optimization and microstructural evolution collectively govern the tribological response of hybrid metal matrix composites. Although morphological features are correlated with wear behaviour, quantitative surface characterization (e.g., roughness parameters or 3D profilometry) was not performed in the present study, and therefore the interpretation is based on SEM observations and tribological measurements.

## Conclusions

The Taguchi method was effectively employed to analyse the wear behaviour of both untreated and cryogenically treated specimens. For untreated specimens, load was identified as the most influential parameter affecting wear scar depth, contributing 44.53%, followed by time (19.05%), reinforcement (18.73%), and sliding speed (15.60%). In cryogenically treated specimens, load also remained the dominant factor, contributing 41.74%, while time, reinforcement, and speed contributed 20.11%, 19.06%, and 16.79%, respectively. A similar trend was observed for the coefficient of friction. In untreated specimens, load exerted the highest influence (39.98%), followed by time (23.45%), reinforcement (16.58%), and speed (15.51%). For cryogenically treated specimens, load showed an even greater contribution of 52.83%, whereas time, reinforcement, and speed accounted for 17.31%, 10.22%, and 3.99%, respectively. The improved tribological performance can be attributed to the synergistic interaction between SiC as a load-bearing reinforcement and graphite as a solid lubricant forming a protective tribofilm during sliding. The multiple regression model developed using ANOVA established a satisfactory correlation for evaluating the wear behaviour of the hybrid combinations of both untreated and cryogenically treated specimens, demonstrating reasonable predictive capability. Cryogenic treatment resulted in a reduction in wear scar depth and coefficient of friction under the tested conditions. Among the compositions investigated, Al6061 reinforced with 13% SiC and 2% Gr exhibited relatively improved wear performance within the experimental range considered. The findings are limited to the experimental conditions considered, and further investigation incorporating mechanical property evaluation and extended validation is recommended. Furthermore, the use of a 90% confidence level, although appropriate for the present experimental design, provides a less stringent criterion for statistical significance than the conventional 95% confidence level and should therefore be interpreted with due consideration.

## Data Availability

Data sets generated during the current study are available from the corresponding author on reasonable request.
